# Supplementary material for: Activation and Identification of a Griseusin Cluster in Streptomyces sp. CA-256286 by Employing Transcriptional Regulators and Multi-Omics Methods
Source: Molecules. 2021 Oct 30;26(21):6580. doi: 10.3390/molecules26216580 (PMC8588249; doi:10.3390/molecules26216580)
Supplement: Supplementary file 1 [file molecules-26-06580-s001.zip › molecules-1429064-Supplementary.pdf]

Activation and identification of a griseusin cluster in *Streptomyces* sp. CA-256286 by employing transcriptional regulators and multi-omics methods

Charlotte Beck[1†], Tetiana Gren[1†], Francisco Javier Ortiz-López[2†], Tue Sparholt Jørgensen[1], Daniel Carretero-Molina[2], Jesús Martín Serrano[2], José R. Tormo[2], Daniel Oves-Costales[2], Eftychia E. Kontou[1], Omkar S. Mohite[1], Erik Mingyar[3,4], Evi Stegmann[3,4], Olga Genilloud[2*] and Tilmann Weber[1*]

Affiliations

1. The Novo Nordisk Foundation Center for Biosustainability, Technical University of Denmark, Kemitorvet, Building 220, 2800 Kgs. Lyngby, Denmark; chabec@biosustain.dtu.dk (C.B.); tetgre@biosustain.dtu.dk (T.G.); tuspjo@biosustain.dtu.dk (T.S.J.); eeko@biosustain.dtu.dk (E.E.K.); omkmoh@biosustain.dtu.dk (O.S.M.)
2. Fundación MEDINA, Centro de Excelencia en Investigación de Medicamentos Innovadores en Andalucía, Parque Tecnológico de Ciencias de la Salud, Av. Conocimiento, 34, 18016 Granada, Spain ; ja-vier.ortiz@medinaandalucia.es (F.J.O.-L.); daniel.carretero@medinaandalucia.es (D.C.-M.); je-sus.martin@medinaandalucia.es (J.M.S.); ruben.tormo@medinaandalucia.es (J.R.T.); da-niel.oves@medinaandalucia.es (D.O.-C.)
3. Department of Microbial Bioactive Compounds, Interfaculty Institute of Microbiology and Infection Medicine, Eberhard Karls Universität Tübingen, Auf der Morgenstelle 28, 72076 Tübingen, Germany; erik.mingyar@gmail.com (E.M.); evi.stegmann@biotech.uni-tuebingen.de (E.S.)
4. German Center for Infection Research (DZIF), Partner Site Tübingen, 72076 Tübingen, Germany

* Correspondence: olga.genilloud@medinaandalucia.es (O.G.); tiwe@biosustain.dtu.dk (T.W.); Tel.: +45-24-89-61-32 (T.W.)

† Author contributed equally to the study.

Supplementary Information

Supplementary information 1: Structure elucidation 1
Supplementary information 2: Bioactivity testing 19
Supplementary information 3: antiSMASH prediction 20
Supplementary information 4: Replicates and samples for omics analyses 21
Supplementary information 5: Transcriptomics 22
Supplementary information 6: LC-MS analyses 23
Supplementary information 7: BLAST analyses 31
Supplementary information 8: Biosynthesis of griseusins 32
Supplementary information 9: BGC 1.31 33

Supplementary information 1: Structure elucidation

a

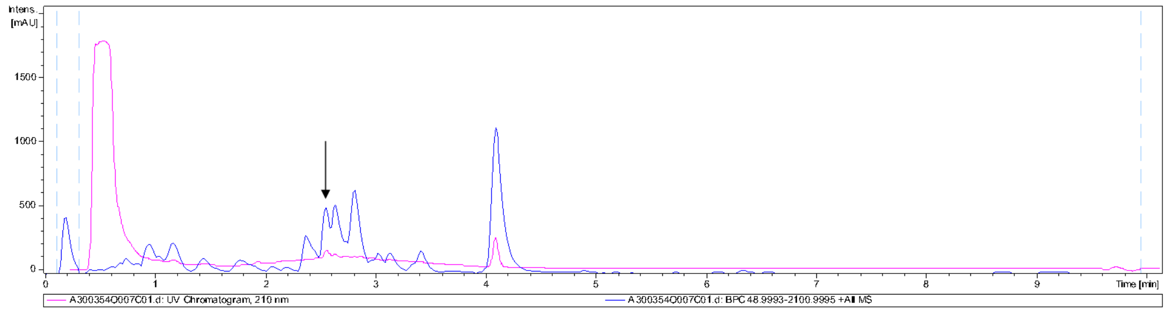

b

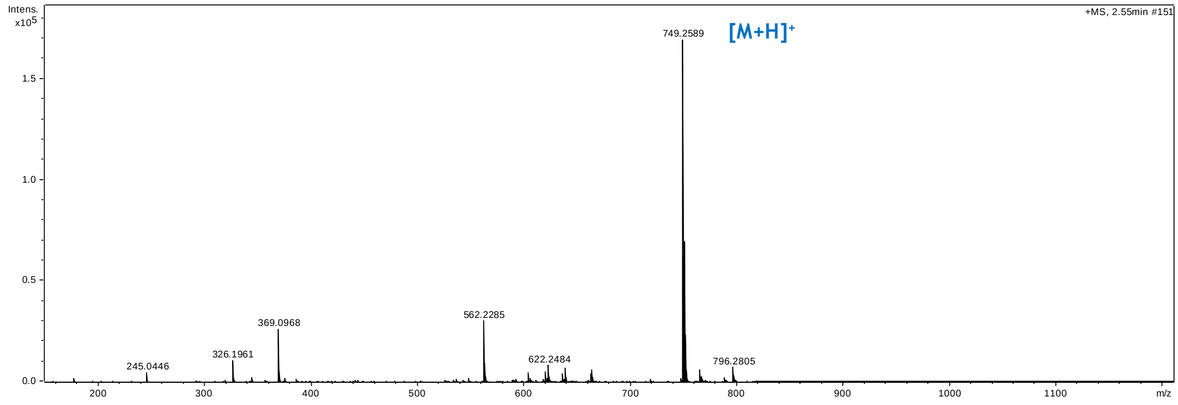

c

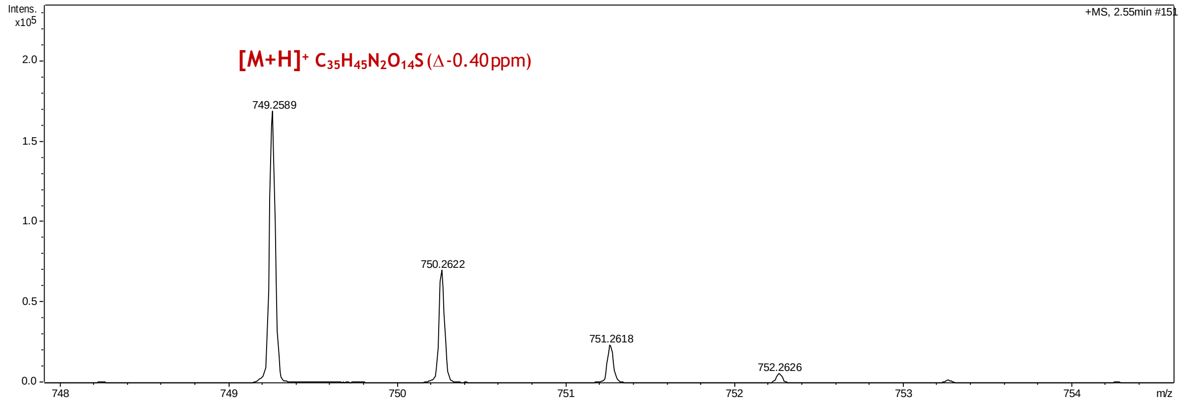

Figure S1: **a)** LC-UV-HRMS chromatogram (UV 210 nm: pink trace; MS+: blue trace) of culture broth extract of CA-300354 (*Streptomyces* sp. CA-256286 with pRM4-SARPs) in MO16 medium. **b)** HRESIMS(+)-TOF spectrum at rt: 2.55 min. **c)** $[M+H]^+$ adduct and molecular formula of **1**

a

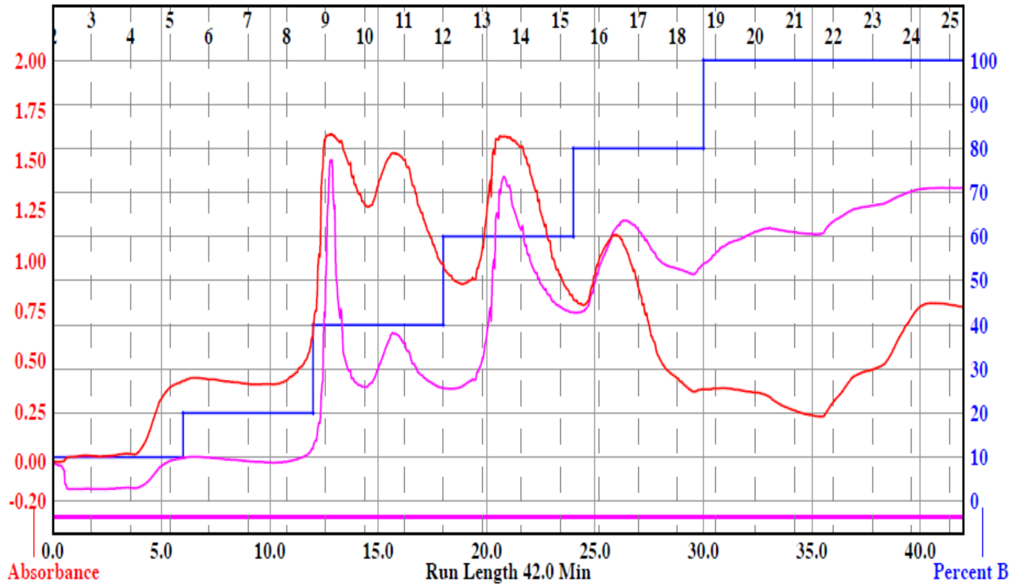

b

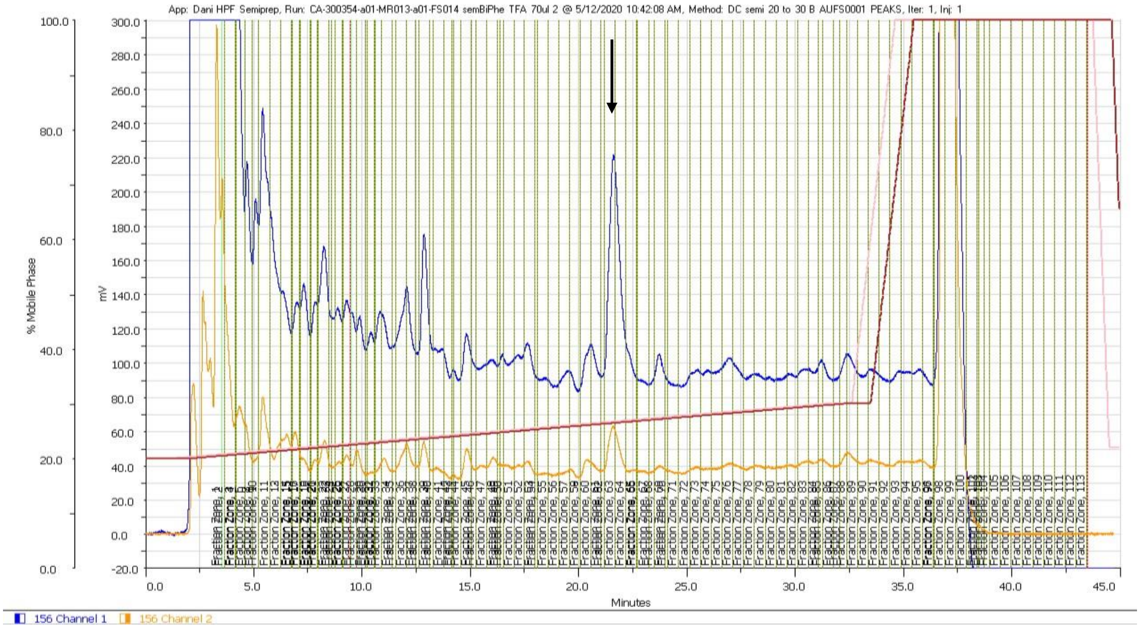

Figure S2: Targeted isolation of **1** (M+H: m/z 749.26) of culture broth extracts of CA-300354 (*Streptomyces* sp. CA-256286 with pRM4-SARPs) regrowth. **a)** MPLC chromatogram. **b)** Further purification by semipreparative HPLC

a

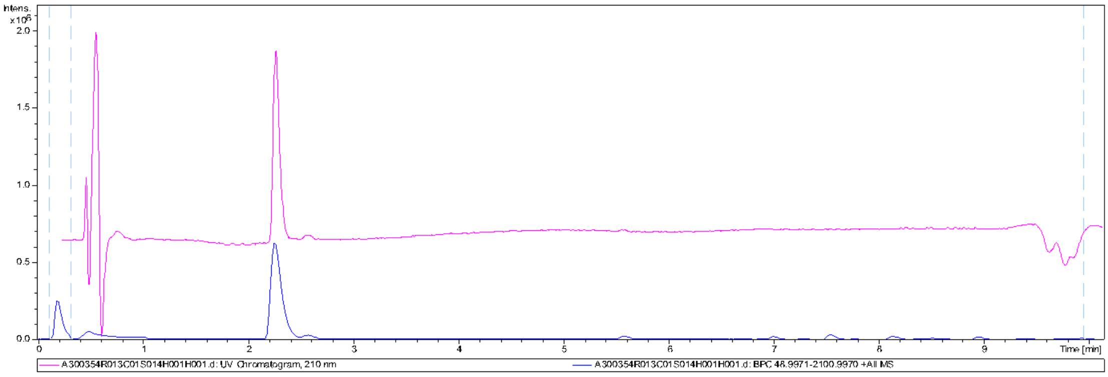

b

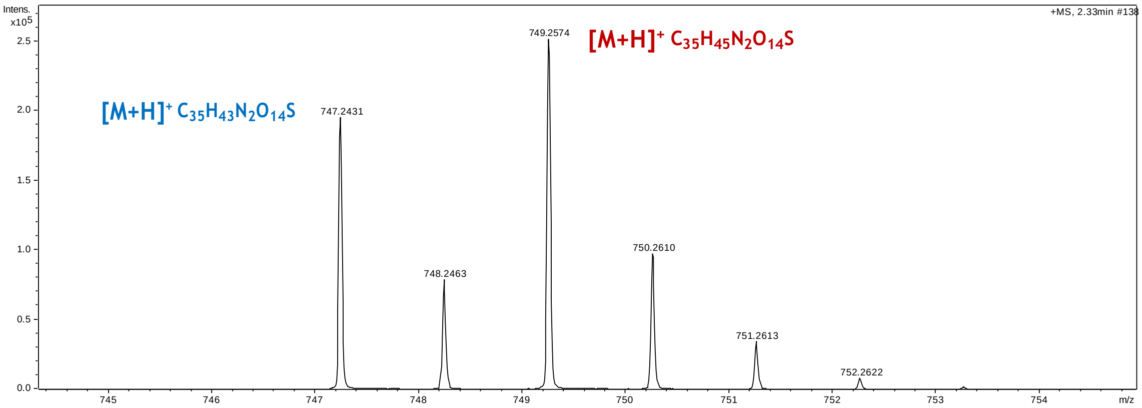

Figure S3: **a)** LC-UV-HRMS chromatogram (UV 210 nm: pink trace; MS+: blue trace) of peak collected by targeted isolation. **b)** HRESIMS(+)-TOF spectrum of peak collected by targeted isolation revelaed the presence of two species, the compound **1** and its oxidized version **2.**

a

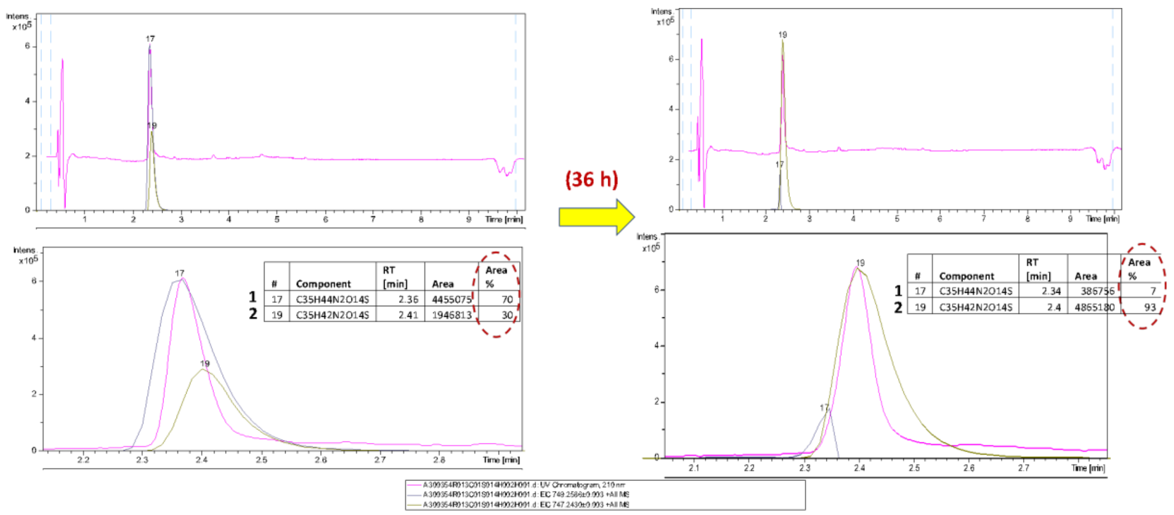

b

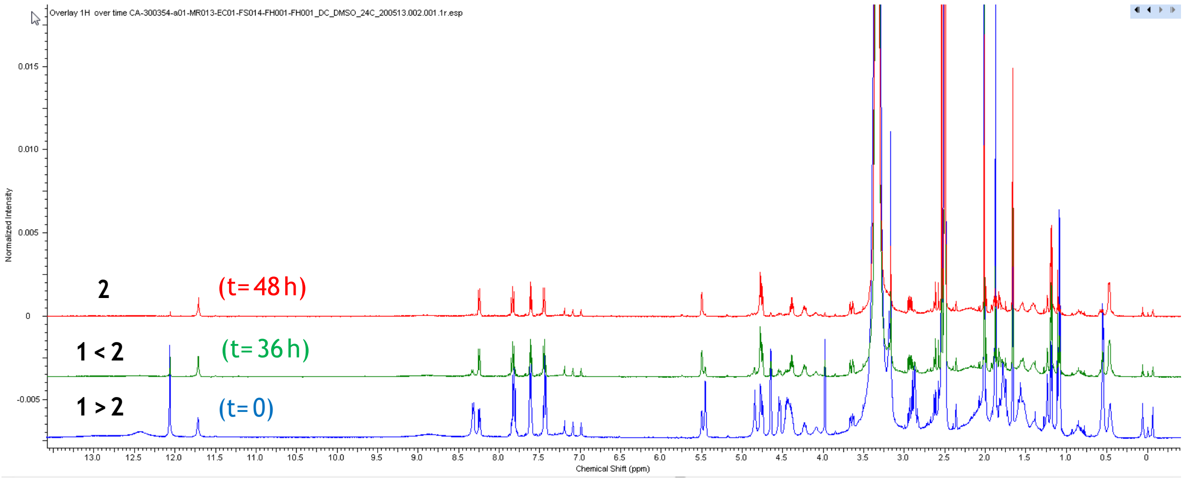

Figure S4: Conversion over time of **1** into **2** (oxidation) monitored by HRMS (**a**) and ^{1}H-NMR (DMSO-*d6*, 500 MHz) (**b**)

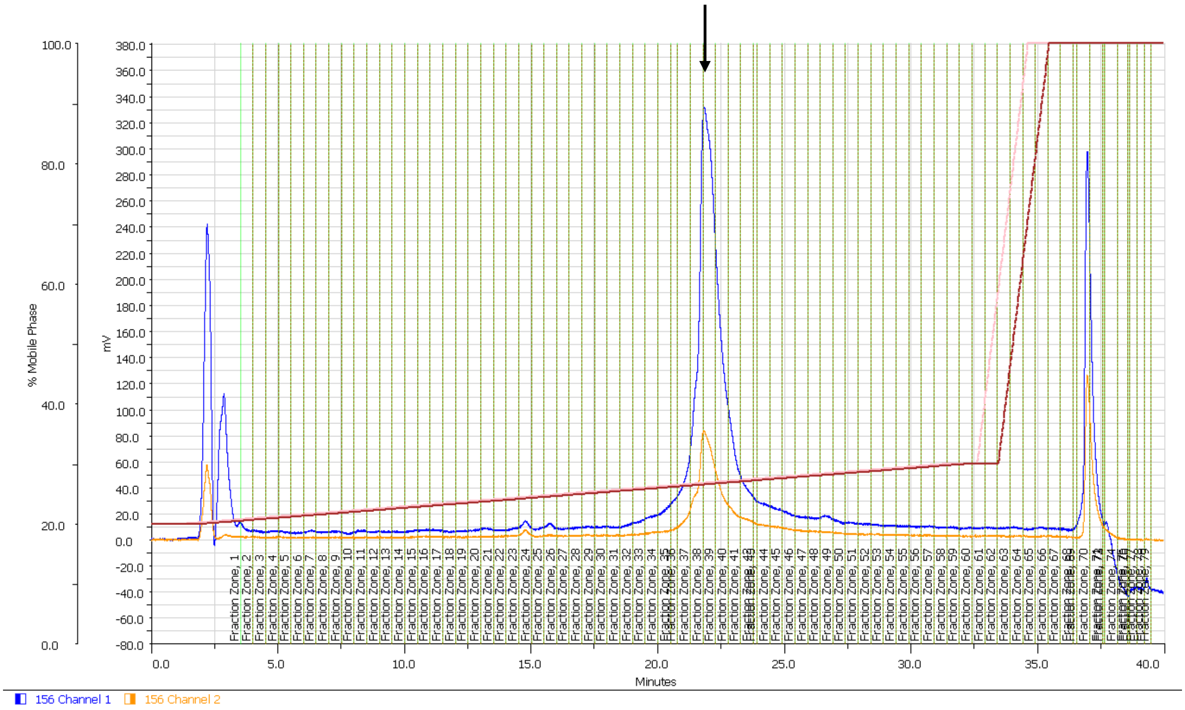

Figure S5: Scale-up purification of **2** by semipreparative HPLC

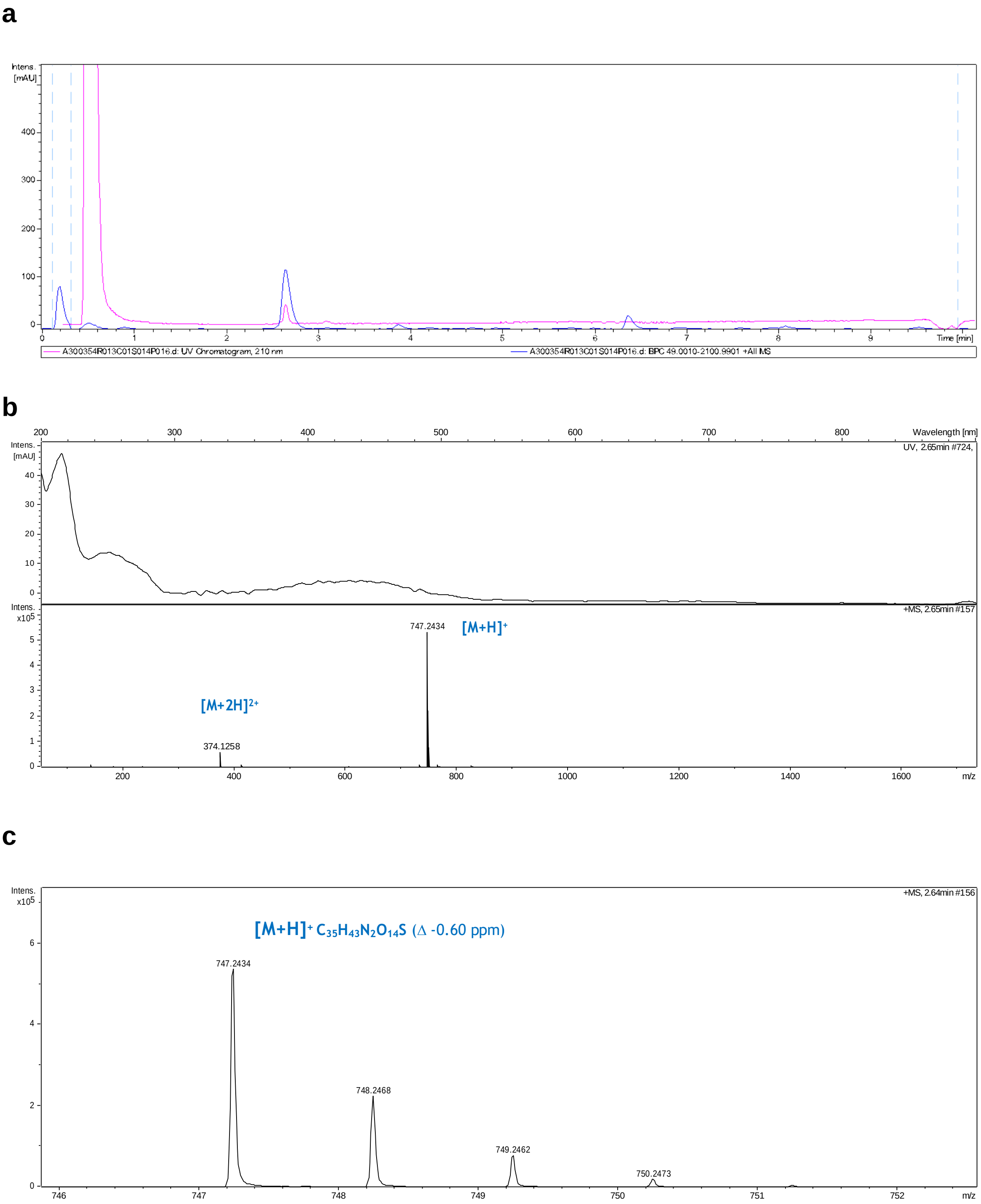

Figure S6: **a)** LC-UV-HRMS chromatogram (UV 210 nm: pink trace; MS+: blue trace) of pure **2**. **b)** HRESIMS(+)-TOF spectrum of pure **2**. **c)** $[M+H]^+$ adduct and molecular formula of **2**.

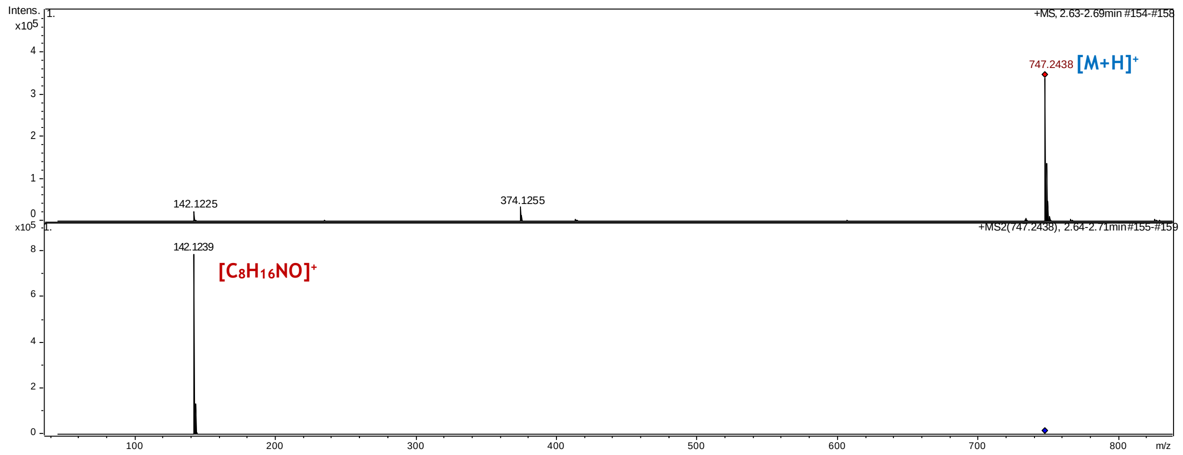

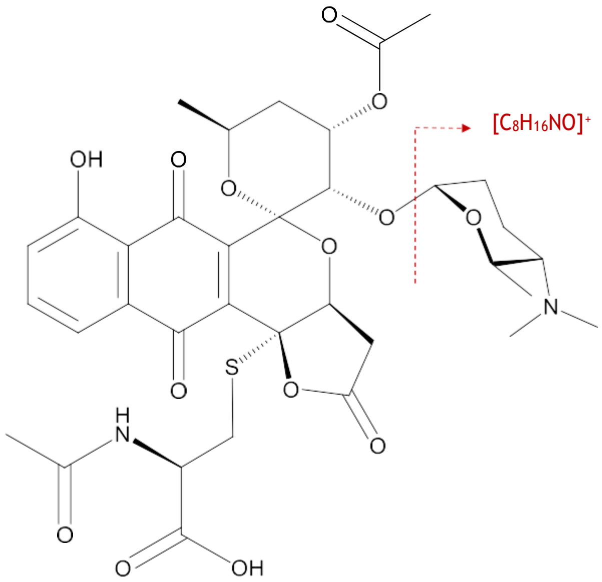

Figure S7: MS/MS fragmentation of $[M+H]^+$ adduct of **2**. Final structure of **2** and the loss of forosamine moiety are shown

a

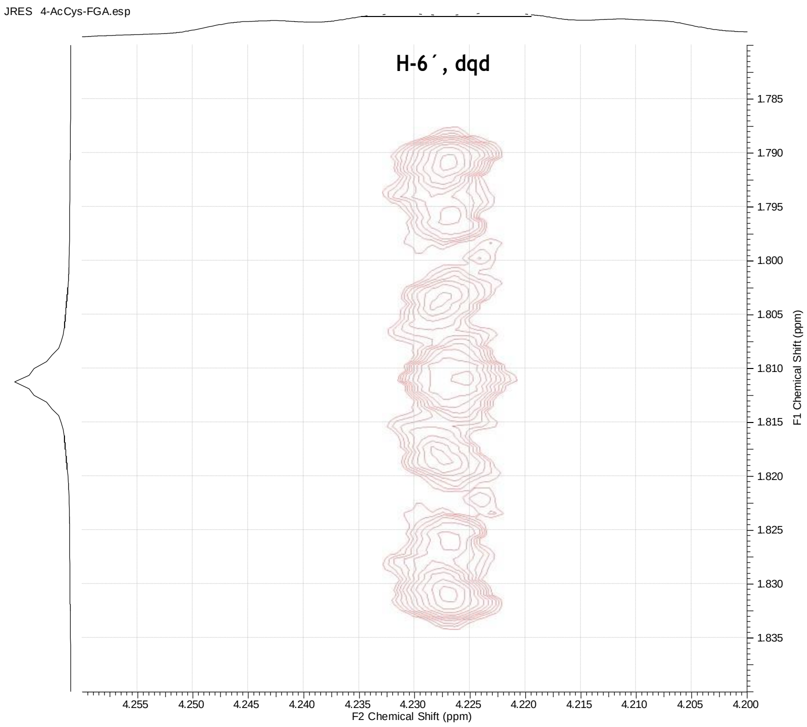

b

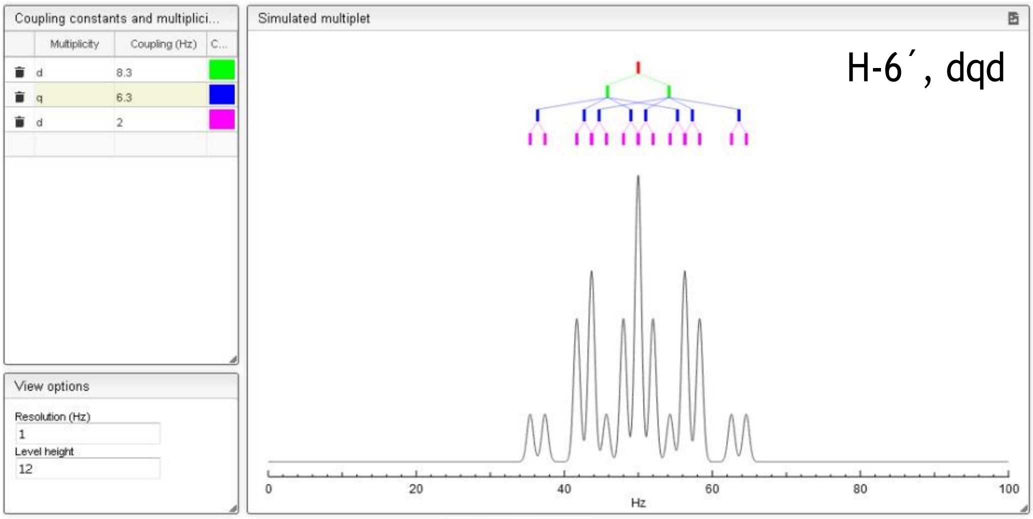

Figure S8: a) H-6´ proton signal in JRES spectrum of **2**. **b**) Multiplet simulation [1] for H-6´ (applied values for ^{1}H-^{1}H coupling constants are indicated) showing good fitting with the multiplicity experimentally observed.

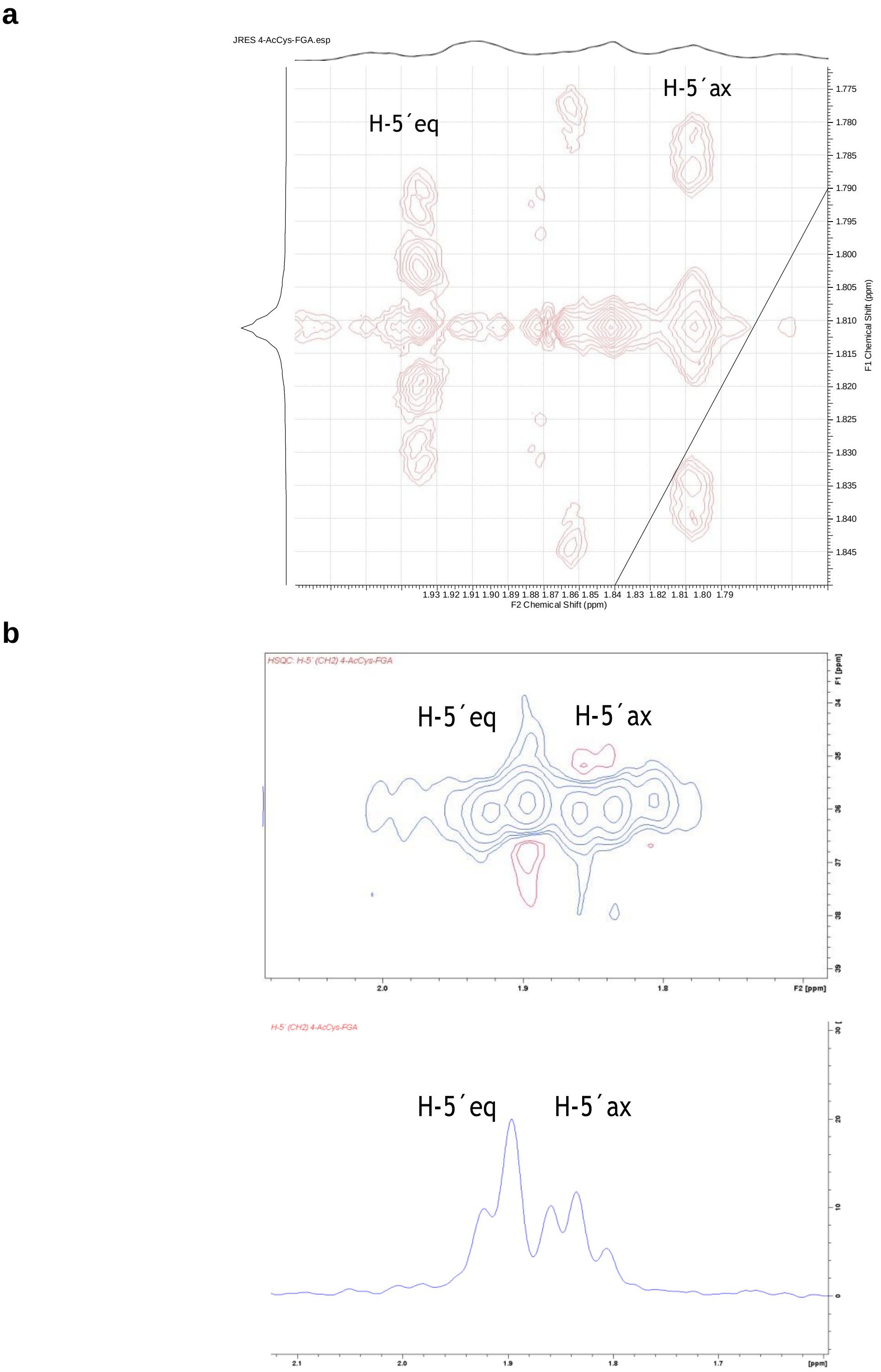

Figure S9: a) H-5´eq and H-5´ax methylene proton signals in JRES spectrum of **2**. **b)** Horizontal HSQC traces for H-5´eq and H-5´´ax protons in **2**, obtained after Gaussian multiplication.[2]. Although ^{1}H-^{1}H coupling constants could not be accurately measured, the proton with higher multiplicity was reasonably assigned to H-5´ ax.

a

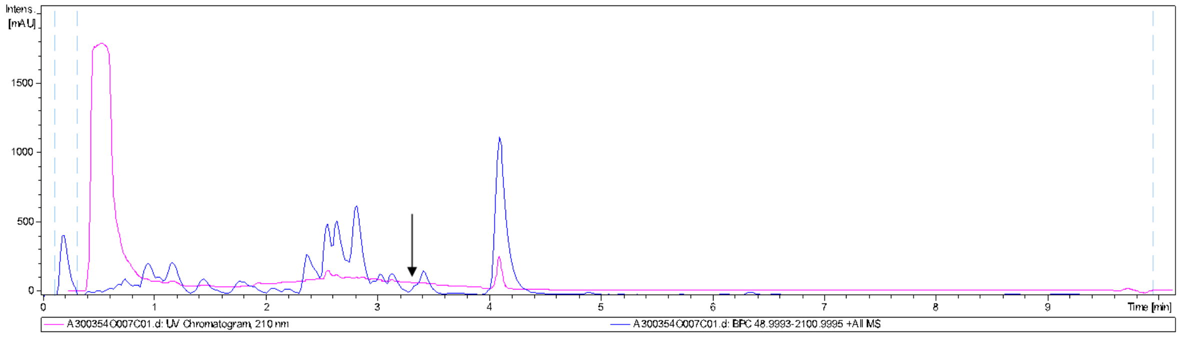

b

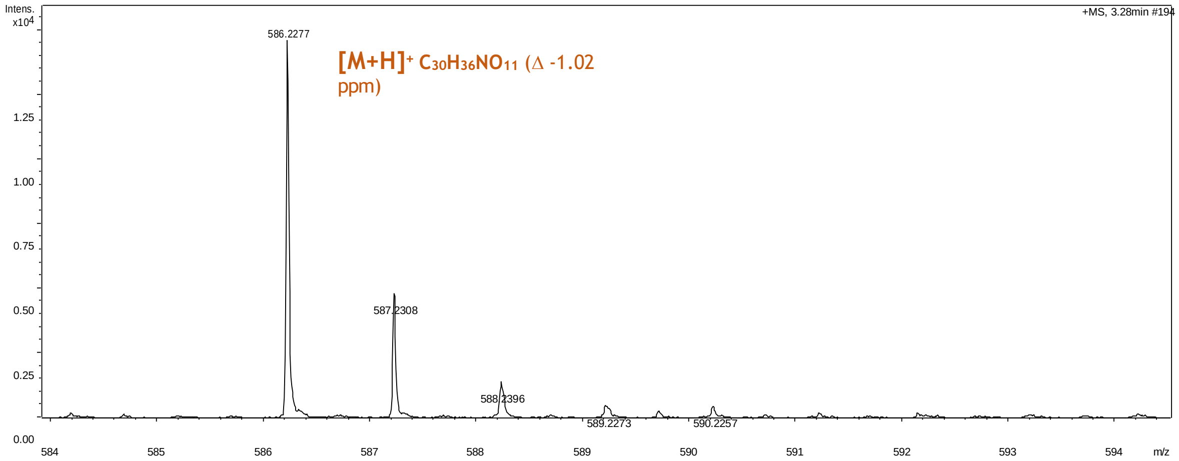

Figure S10: LC-UV-HRMS chromatogram (UV 210 nm: pink trace; MS+: blue trace) of culture broth extract of CA-300354 (*Streptomyces* sp. CA-256286 with pRM4-SARPs) in MO16 medium. **b)** HRESIMS(+)-TOF spectrum at rt: 3.28 min., $[M+H]^+$ adduct and molecular formula of **3**

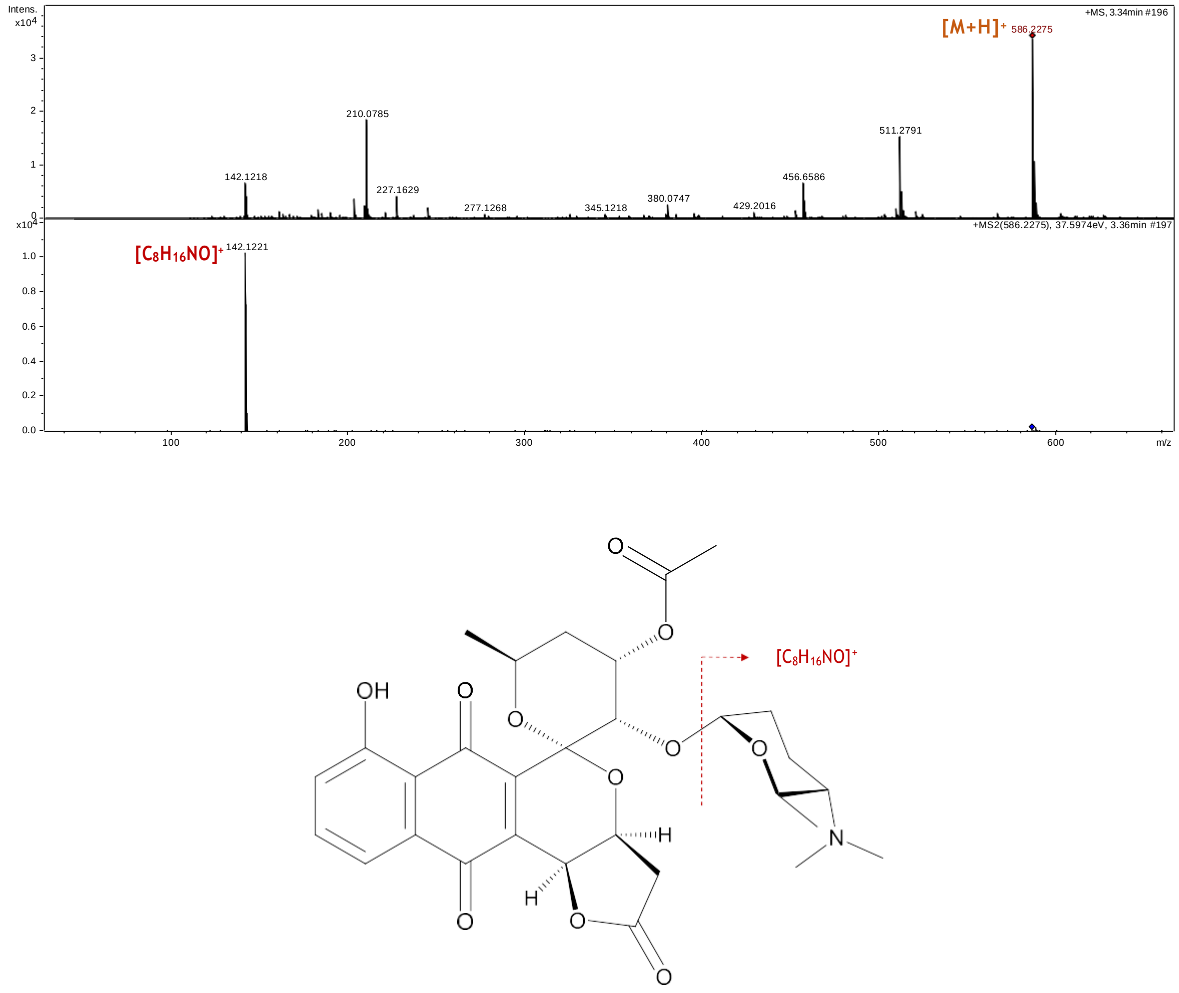

Figure S11: MS/MS fragmentation of $[M+H]^+$ adduct of **3**. The loss of forosamine moiety is shown.

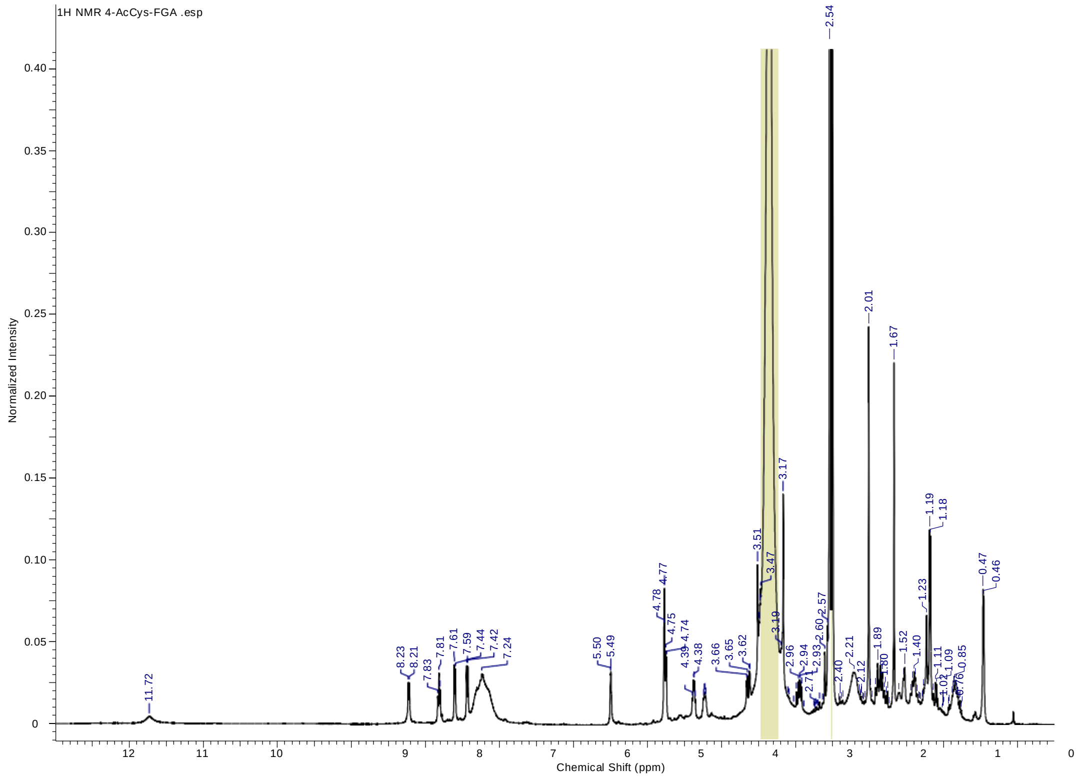

Figure S12: ^{1}H-NMR spectrum (DMSO-*d6*, 500 MHz) of 4-AcCys-FGA (**2**)

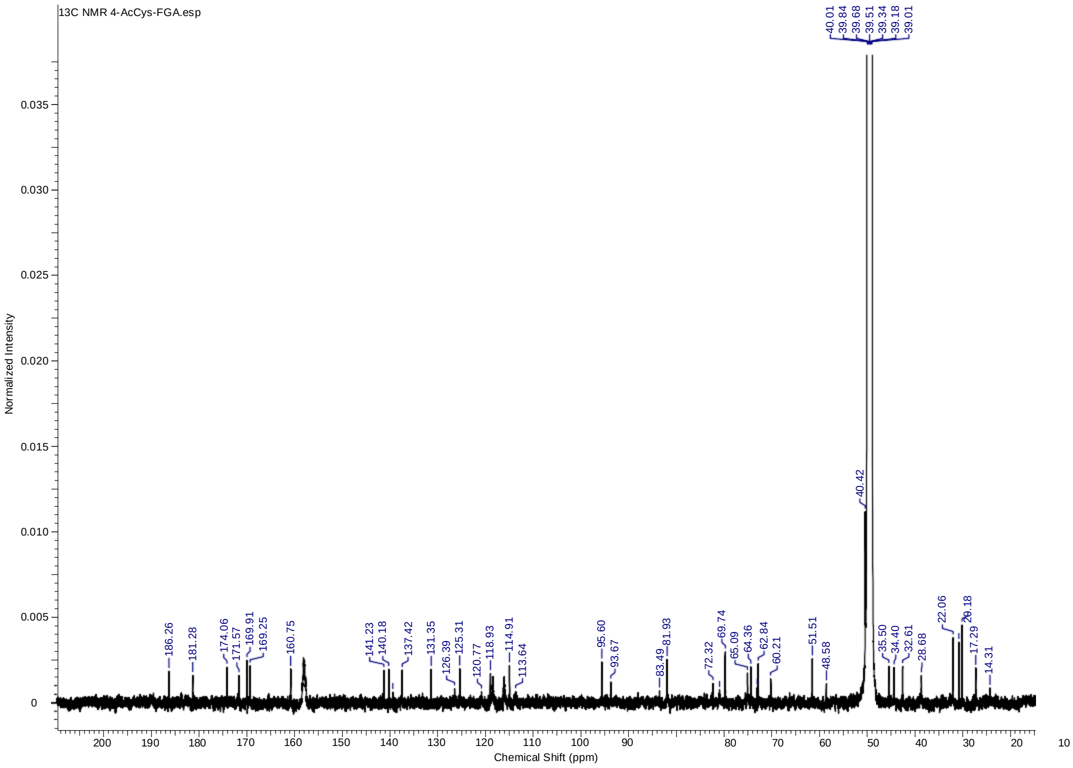

Figure S13: ^{13}C-NMR spectrum (DMSO-*d6*, 500 MHz) of 4-AcCys-FGA (**2**)

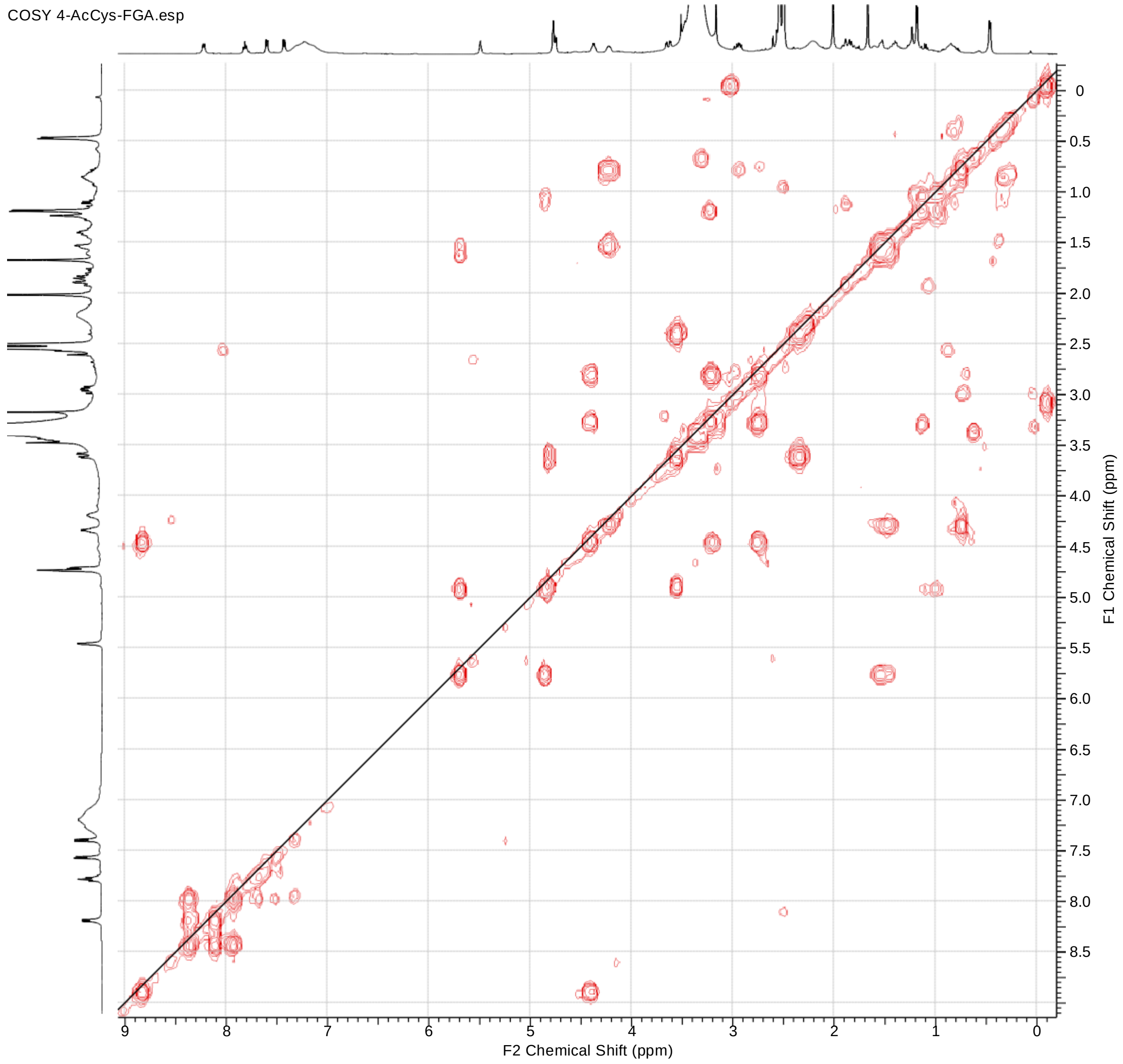

Figure S14: COSY spectrum (DMSO-*d6*, 500 MHz) of 4-AcCys-FGA (**2**)

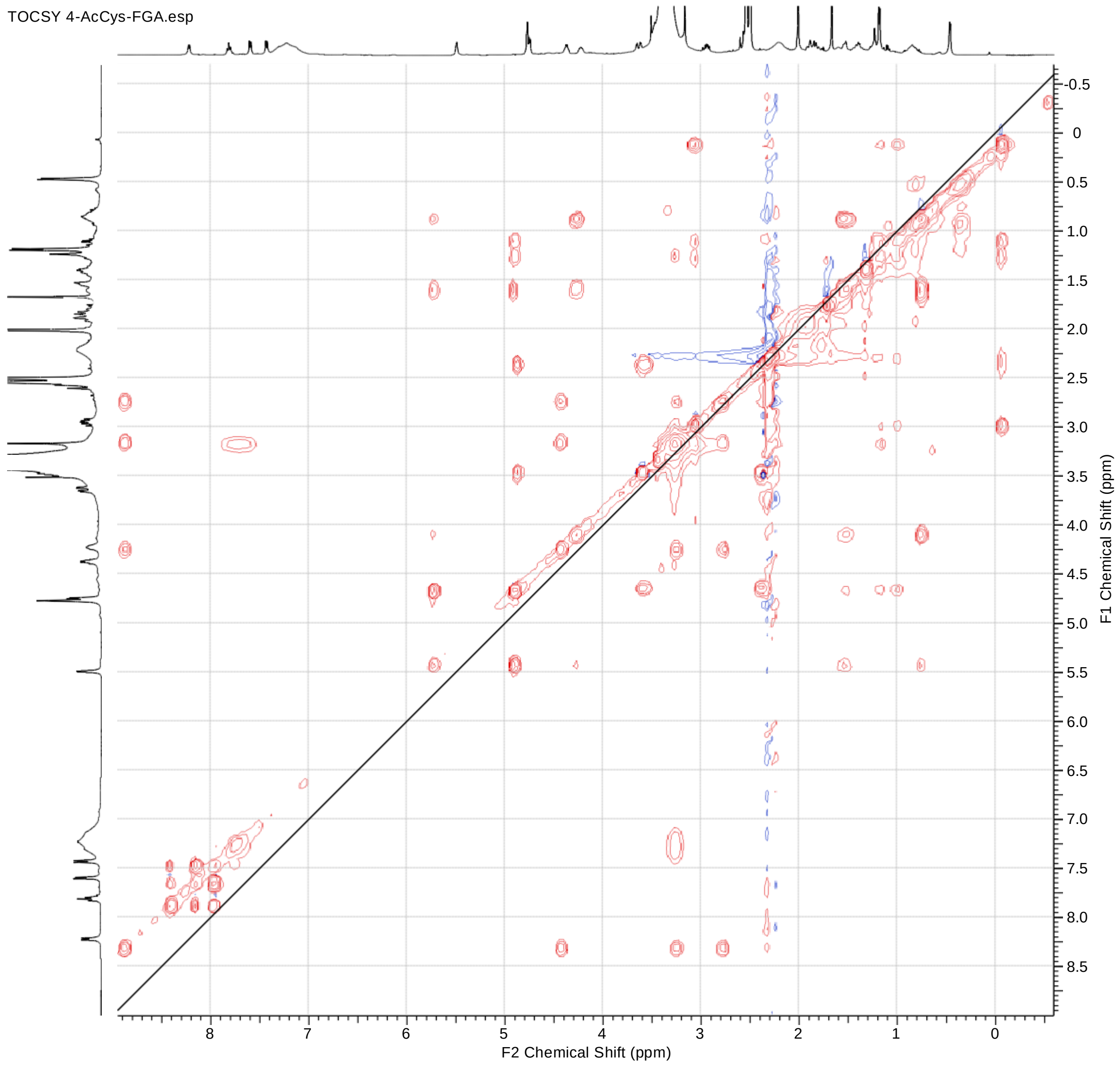

Figure S15: TOCSY spectrum (DMSO-*d6*, 500 MHz) of 4-AcCys-FGA (**2**)

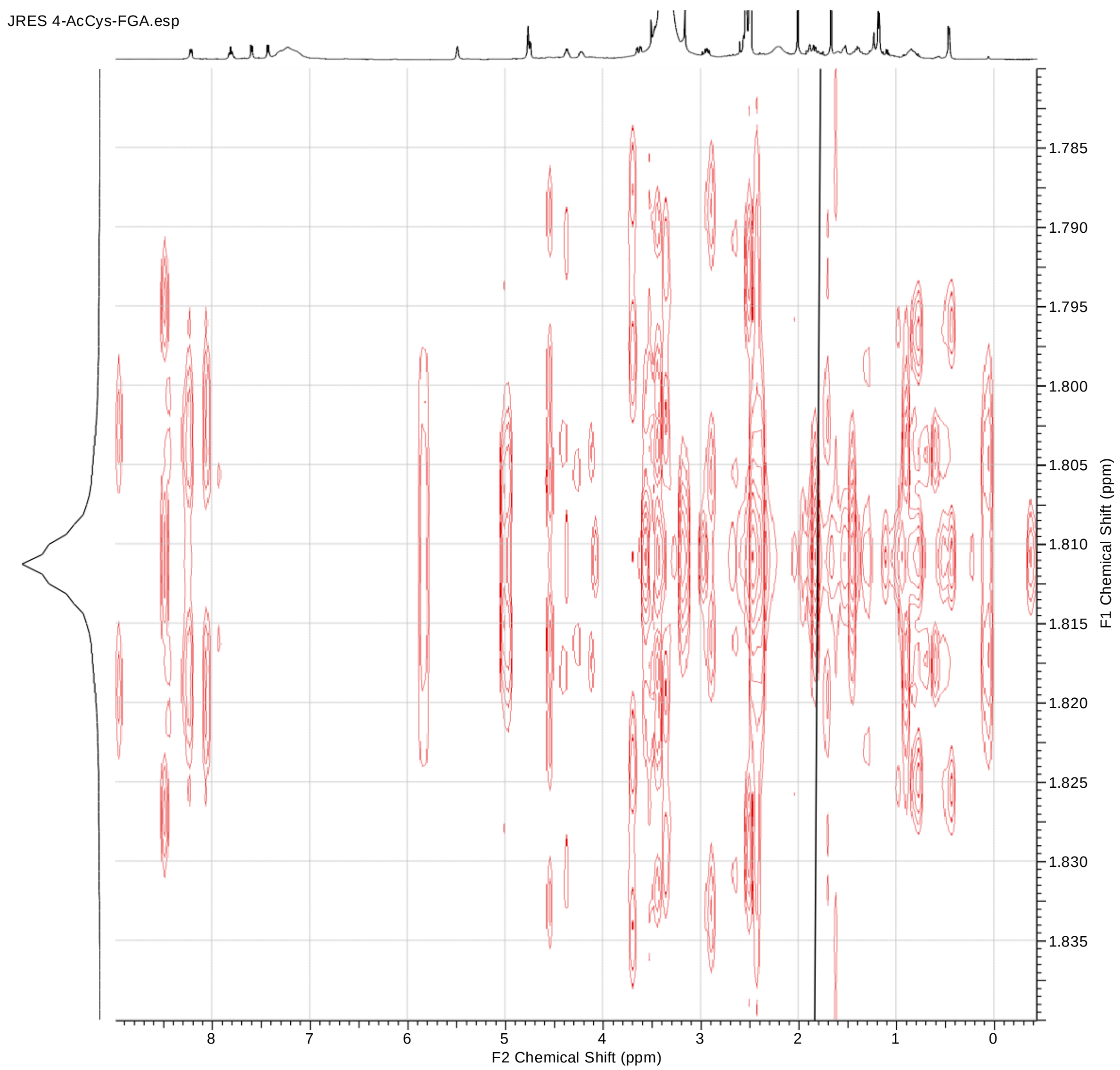

Figure S16: JRES spectrum (DMSO-*d6*, 500 MHz) of 4-AcCys-FGA (**2**)

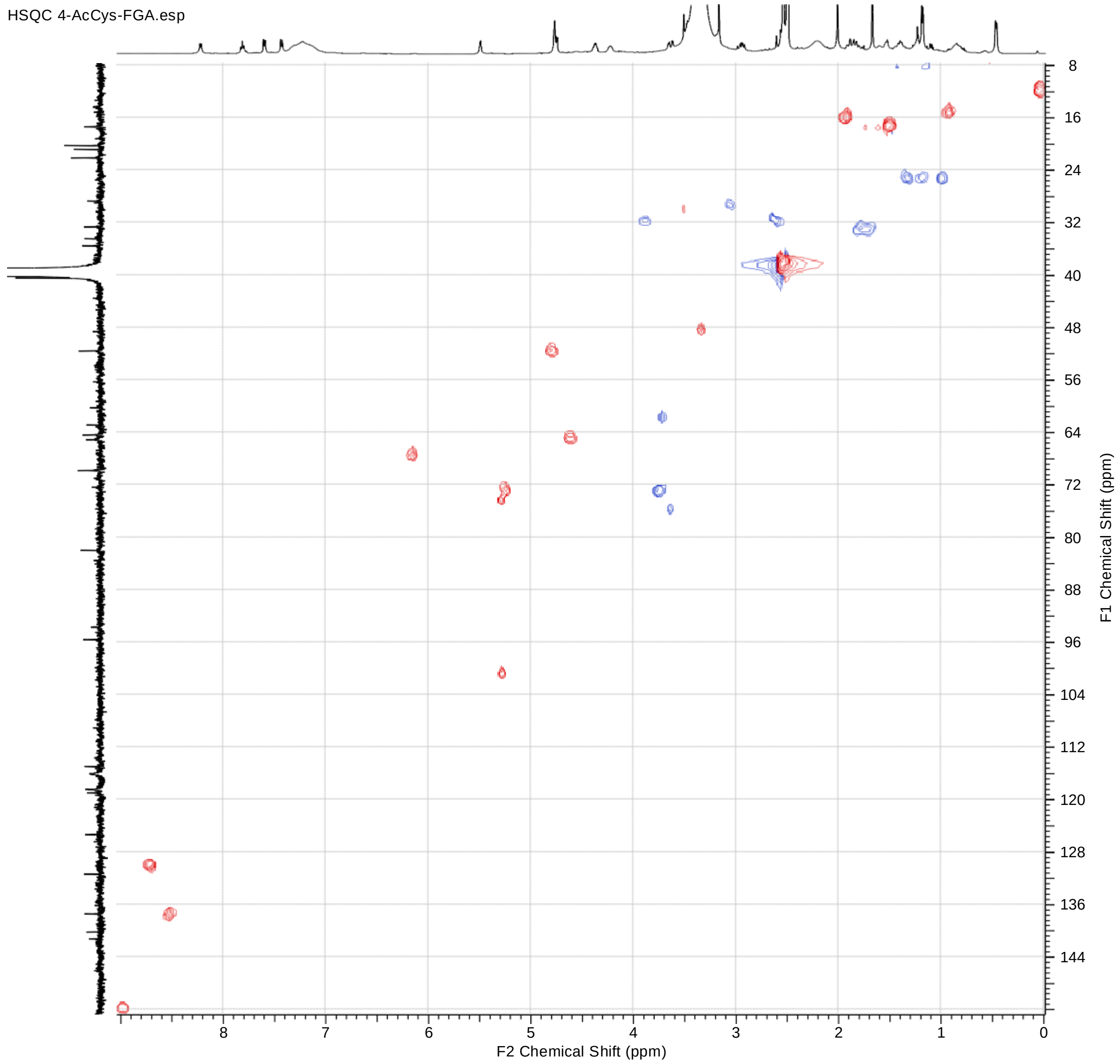

Figure S17: HSQC spectrum (DMSO-*d6*, 500 MHz) of 4-AcCys-FGA (**2**)

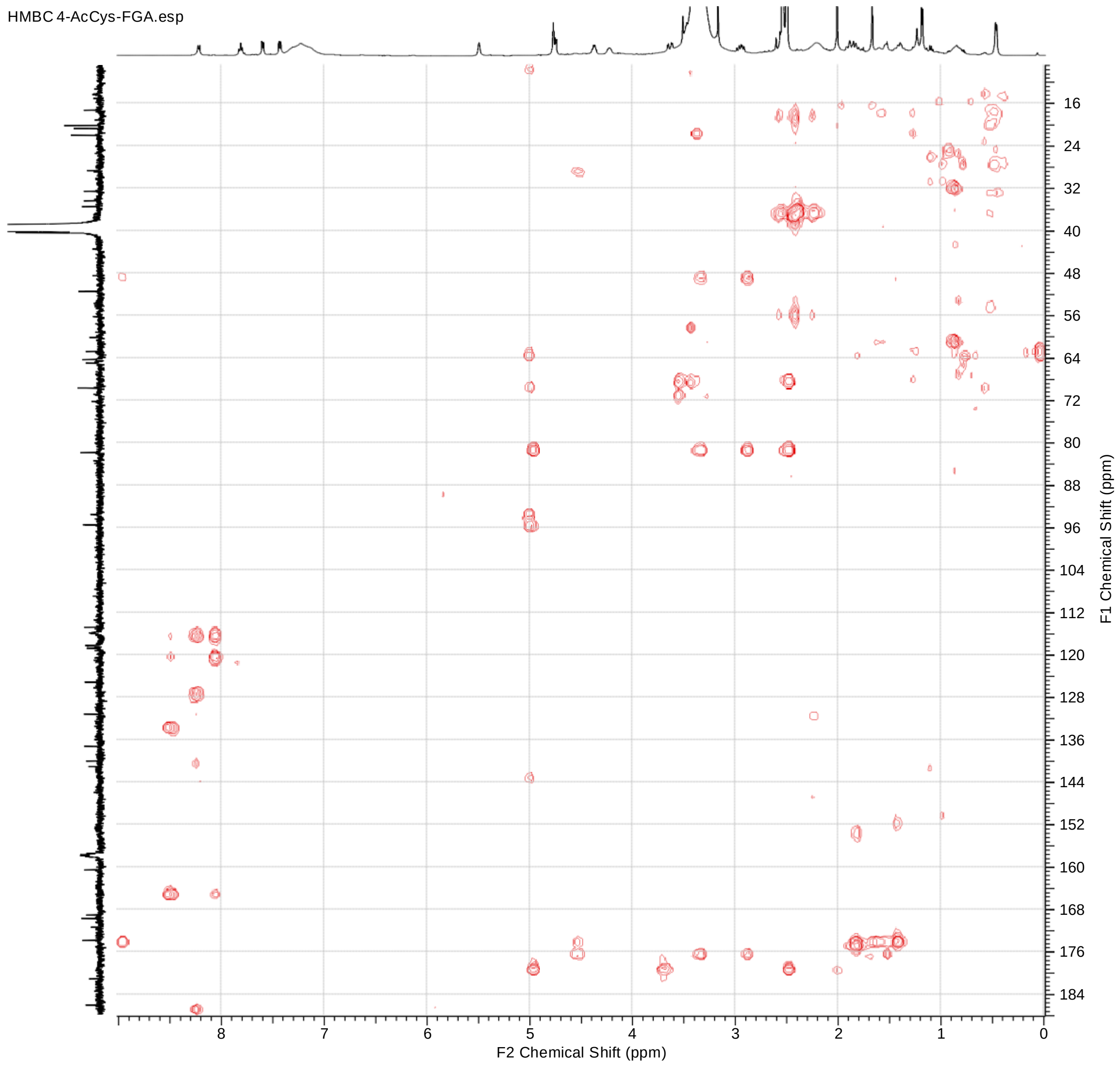

Figure S18: HMBC spectrum (DMSO-*d6*, 500 MHz) of 4-AcCys-FGA (**2**)

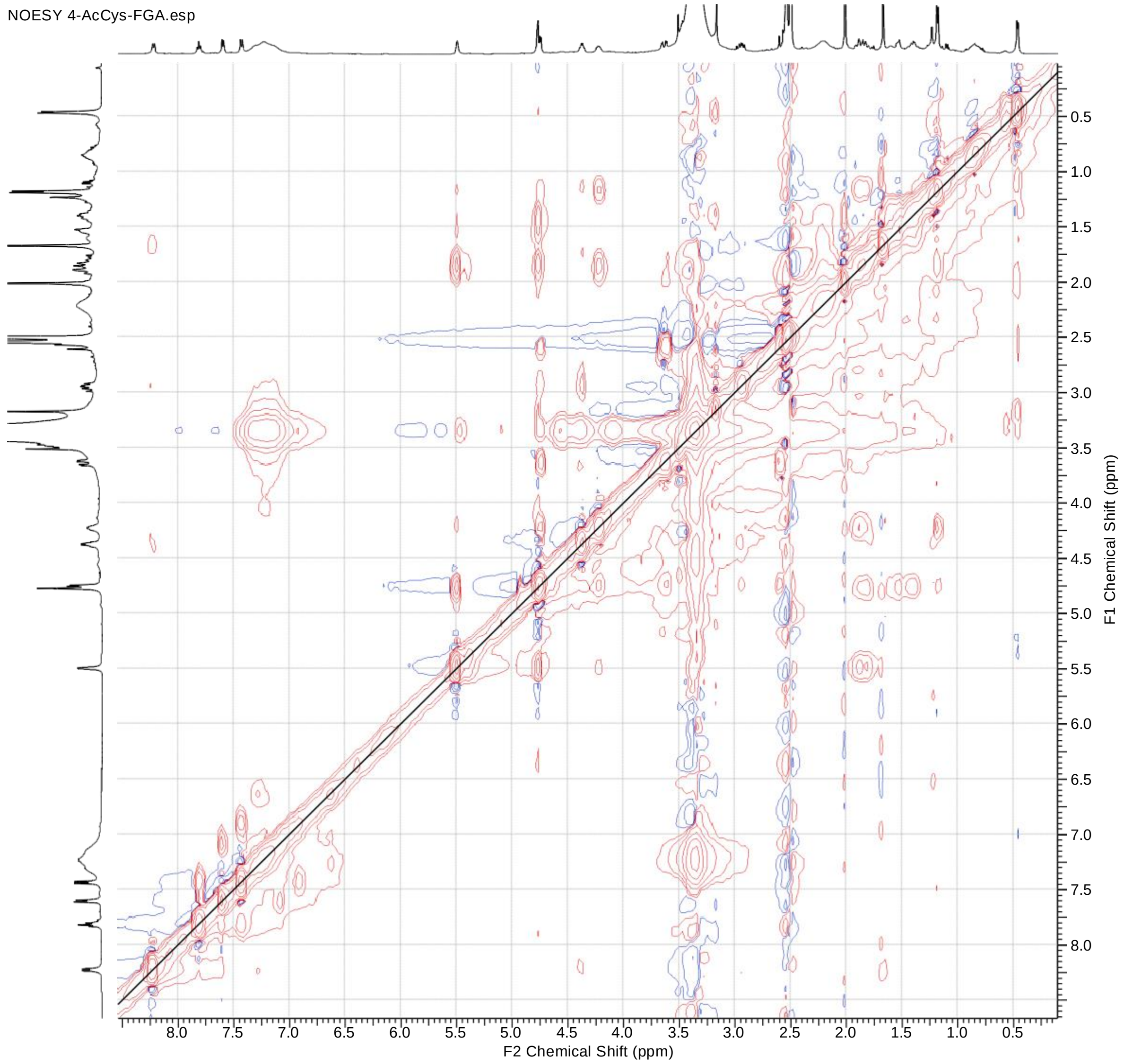

Figure S19: NOESY spectrum (DMSO-*d6*, 500 MHz) of 4-AcCys-FGA (**2**)

References

1. http://www.cheminfo.org/Spectra/NMR/Tools/Multiplet_simulator/index.html.
2. *T. D. W. Claridge, in High-Resolution NMR Techniques in Organic Chemistry, Elsevier, Oxford, 3rd edn, 2016, ch. 3, pp. 86–87.*;

Supplementary information 2: Bioactivity testing

					MSSA ATCC29213						MRSA MB5393					
Medina Sample ID	Row	Column	Compound ID	Conc. (µg/mL)	% INH_1:	% INH_1:	% INH_1:	Average	STDV	MIC (µg/mL)	% INH_1:	% INH_1:	% INH_1:	Average	STDV	MIC (µg/mL)
PL-HPP-000173-A1	A	2	CA-300354-a01-MR013-EC01-FS014-FP017-a01	128	13	3	4	6	5	> 128	7	6	7	7	1	> 128
				64	13	-3	4	4	8		5	5	5	5	0	
				32	6	-6	12	4	9		4	5	4	4	1	
				16	11	-2	9	6	7		4	5	2	4	2	
				8	10	-6	30	11	19		2	9	-2	3	5	
				4	14	-5	6	5	10		0	1	1	1	1	
				2	10	31	6	15	13		2	3	2	2	0	
				1	10	-6	9	4	9		1	5	5	4	2	
				0.5	8	-18	16	2	18		3	4	4	4	0	
				0.25	10	-8	10	4	10		8	3	4	5	3	

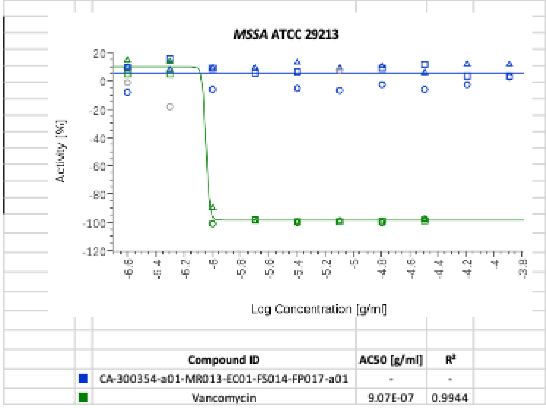

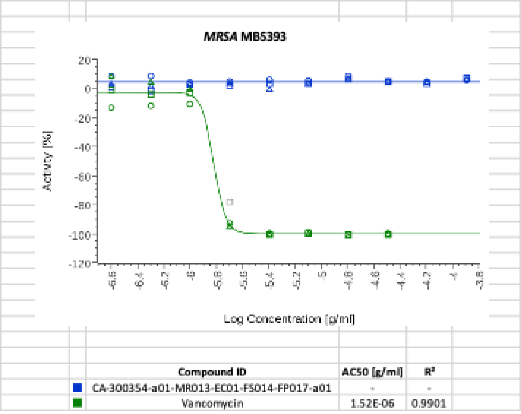

MIC > 128 ug/mL

MIC > 128 ug/mL

Figure S20: Bioactivity testing of AcCys adduct **2** against MSSA and MRSA, showing no activity.

Supplementary information 3: antiSMASH prediction

Region	Type	From	To	Most similar known cluster		Similarity
Region 1.1	NRPS, T1PKS, thiopeptide, LAP	65,689	131,653	lactazole	RiPP:Thiopeptide	33%
Region 1.2	terpene	146,756	170,148	isorenieratene	Terpene	100%
Region 1.3	T3PKS	261,468	302,057	herboxidiene	Polyketide	9%
Region 1.4	melanin	334,998	345,471	melanin	Other	100%
Region 1.5	bacteriocin	528,172	536,427			
Region 1.6	T1PKS, NRPS	550,446	598,095	SGR PTMs	NRP + Polyketide	100%
Region 1.7	linaridin	636,329	656,937	pentostatine / vidarabine	Other	9%
Region 1.8	terpene	680,455	706,615	hopene	Terpene	69%
Region 1.9	NRPS	960,307	1,024,307	nonactin	Polyketide	92%
Region 1.10	arylpolyene, NRPS-like	1,032,426	1,075,207	formicamycins A-M	Polyketide	11%
Region 1.11	NRPS	1,121,481	1,179,892	asukamycin	Polyketide:Type II	12%
Region 1.12	terpene	1,266,611	1,286,145	2-methylisoborneol	Terpene	100%
Region 1.13	bacteriocin	1,304,256	1,315,587			
Region 1.14	butyrolactone	1,476,959	1,486,500			
Region 1.15	siderophore	1,640,684	1,652,888	ficellomycin	NRP	3%
Region 1.16	terpene	2,080,278	2,095,401			
Region 1.17	lanthipeptide	2,434,192	2,455,327	AmfS	RiPP:Lanthipeptide	100%
Region 1.18	T1PKS	2,745,040	2,809,370	argimycin PI / argimycin PII / nigrifactin / argimycin PIV / argimycin PV / argimycin PVI / argimycin PIX	Polyketide:Modular type I	27%
Region 1.19	lassopeptide	3,358,147	3,380,176	keywimysin	RiPP	100%
Region 1.20	T2PKS	3,435,730	3,506,864	prejadomycin / rabelomycin / gaudimycin C / gaudimycin D / UWM6 / gaudimycin A	Polyketide:Type II + Saccharide:Hybrid/tailoring	27%
Region 1.21	betalactone	4,047,261	4,074,934	divergolide A / divergolide B / divergolide C / divergolide D	Polyketide:Modular type I	6%
Region 1.22	NRPS	4,421,087	4,484,173	phosphonoglycans	Saccharide	3%
Region 1.23	thiopeptide, LAP	4,678,585	4,713,148			
Region 1.24	NRPS-like	4,966,191	5,009,033	bottromycin A2	RiPP:Bottromycin	39%
Region 1.25	siderophore	5,095,552	5,107,330	desferrioxamin B	Other	100%
Region 1.26	lanthipeptide	5,165,457	5,188,765			
Region 1.27	NRPS, PKS-like	5,500,642	5,576,586	decaplanin	NRP:Glycopeptide	7%
Region 1.28	lassopeptide	5,751,892	5,774,430	albusnodin	RiPP	100%
Region 1.29	ectoine	6,200,194	6,210,147	ectoine	Other	100%
Region 1.30	terpene	6,670,760	6,689,790	steffimycin D	Polyketide:Type II + Saccharide:Hybrid/tailoring	19%
Region 1.31	T2PKS	6,839,117	6,911,635	hiroshidine	Polyketide	41%
Region 1.32	T3PKS	7,216,064	7,257,182	herboxidiene	Polyketide	6%
Region 1.33	T1PKS, NRPS	7,267,073	7,317,119	arsono-polyketide	Polyketide	45%
Region 1.34	NRPS	7,326,987	7,375,242	coelichelin	NRP	81%
Region 1.35	NRPS, transAT-PKS, T1PKS, PKS-like	7,396,465	7,513,122	streptobactin	NRP	94%
Region 1.36	terpene	7,521,039	7,543,252	geosmin	Terpene	100%
Region 1.37	butyrolactone	7,572,437	7,583,324	coelimycin P1	Polyketide:Modular type I	16%
Region 1.38	ectoine	7,674,984	7,685,385			
Region 2.1	terpene	21,647	42,699	platencin	Terpene	9%
Region 2.2	ectoine	245,190	255,600	ectoine	Other	50%
Region 2.3	T1PKS	302,669	348,989	actinomycin D	NRP	10%

Figure S21: Prediction of biosynthetic regions in *Streptomyces* sp. CA-256286

Supplementary information 4: Replicates and samples for omics analyses

Table S1: Replicates of strains CA-300354 (*Streptomyces* sp. CA-256286 with pRM4-SARPs) and CA-300355 (*Streptomyces* sp. CA-256286 with pRM4) harvested for proteomics and transcriptomics analyses, the number given in RNA purification and sequencing, and which replicates successfully resulted in data.

**Strain**	**Replicate**	**Number in RNA purification**	**Proteomics**	**Transcriptomics**
CA-300354	A	11	Yes	No
CA-300354	B	12	No	Yes
CA-300354	C	13	Yes	Yes
CA-300354	D	14	Yes	Yes
CA-300354	E	15	Yes	Yes
CA-300355	A	16	Yes	Yes
CA-300355	B	17	Yes	Yes
CA-300355	C	18	Yes	Yes
CA-300355	D	19	Yes	Yes
CA-300355	E	20	No	No

Supplementary information 5: Transcriptomics

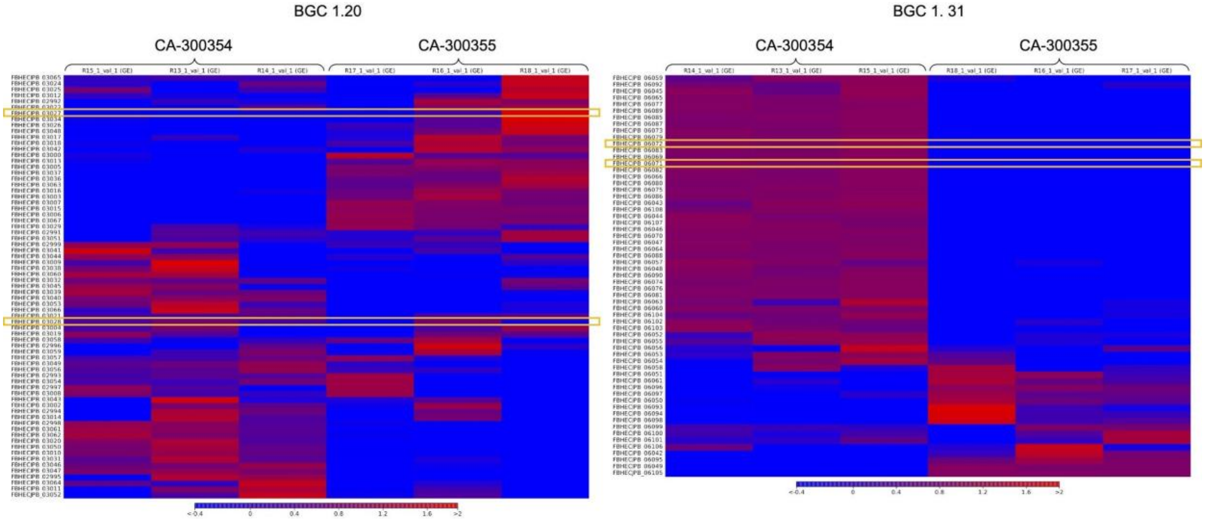

Figure S22: Heat maps of transcriptomics data for three replicates of each strain CA-300354 (*Streptomyces* sp. CA-256286 with pRM4-SARPs) and CA-300355 (*Streptomyces* sp. CA- 256286 with pRM4) for all genes predicted in BGC 1.20 (FBHECJPB_02990 to FBHECJPB_03067) and 1.31 (FBHECJPB_06042 to FBHECJPB_06108). The colors represent expression levels, where red represents high and blue represents low expression. The two core type II PKS genes from each cluster are outlined in yellow. The data was analyzed with CLC Genomics with default parameters.

Supplementary information 6: LC-MS analyses

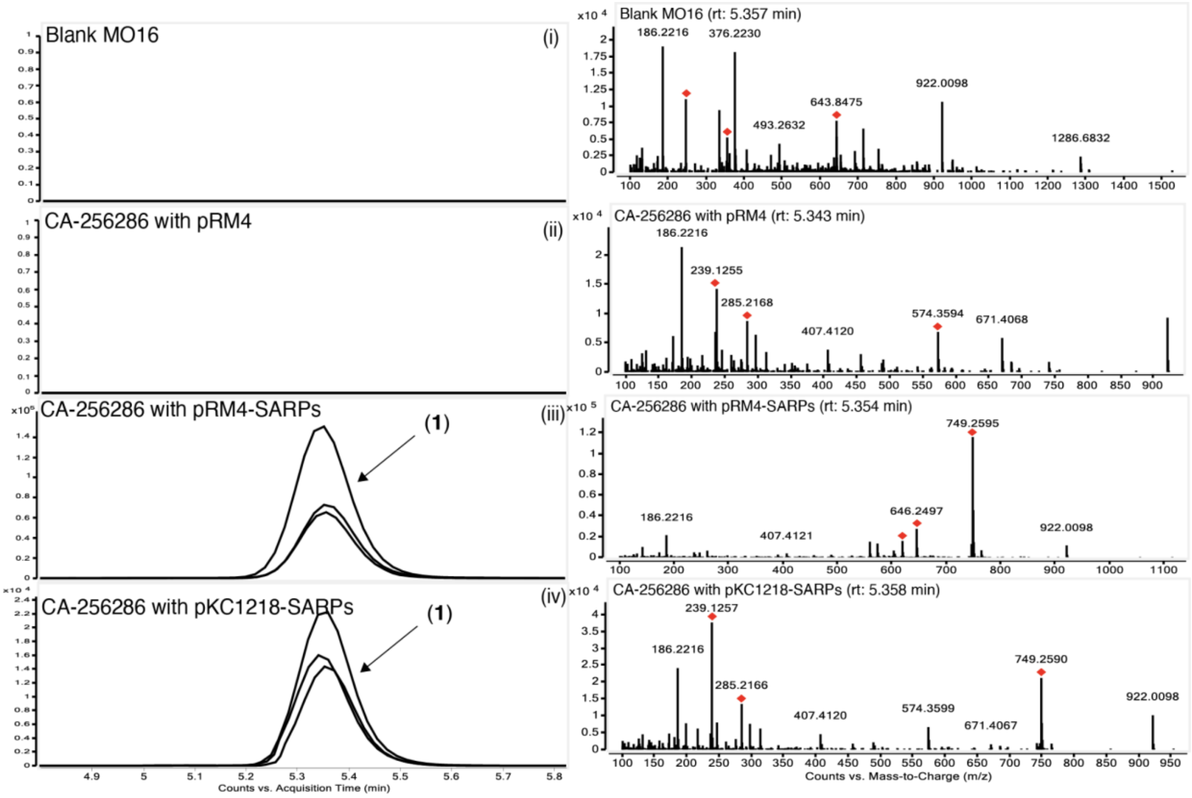

Figure S23: LC-MS analysis for the production of (**1**) in CA-300355 (CA-256286 with pRM4) (ii), CA-300354 (CA-256286 with pRM4-SARPs) (iii) and CA-256286 with pKC1218-SARPs (iv). Chromatogram (i) represents blank samples from MO16 medium (negative control). Replicates are displayed in overlaid mode. On the right from every EIC, a mass spectrum is extracted for approximate RT 5.35 min for one of the replicates.

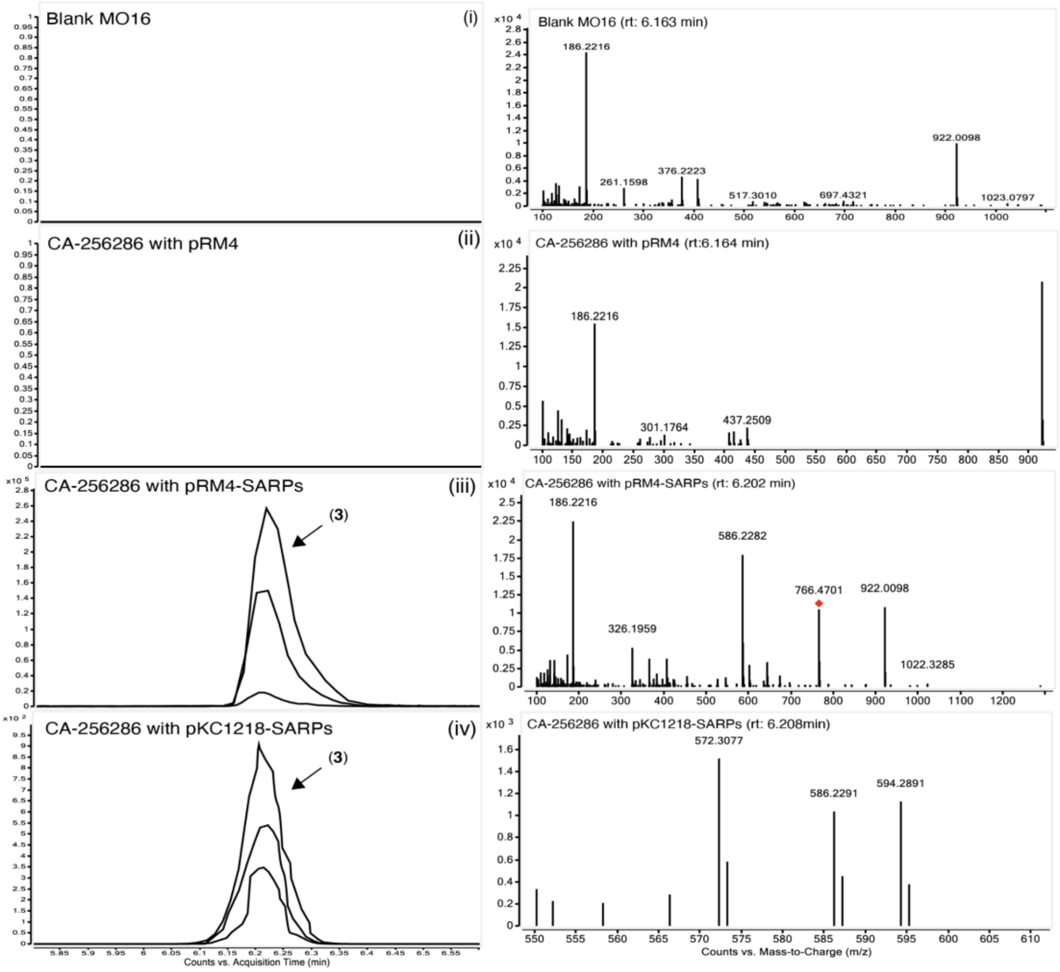

Figure S24: LC-MS analysis for the production of (**3**) in CA-300355 (CA-256286 with pRM4) (ii), CA-300354 (CA-256286 with pRM4-SARPs) (iii) and CA-256286 with pKC1218-SARPs (iv). Chromatogram (i) represents blank samples from MO16 medium (negative control). Replicates are displayed in overlaid mode. On the right from every EIC, a mass spectrum is extracted for approximate RT 6.21 min.

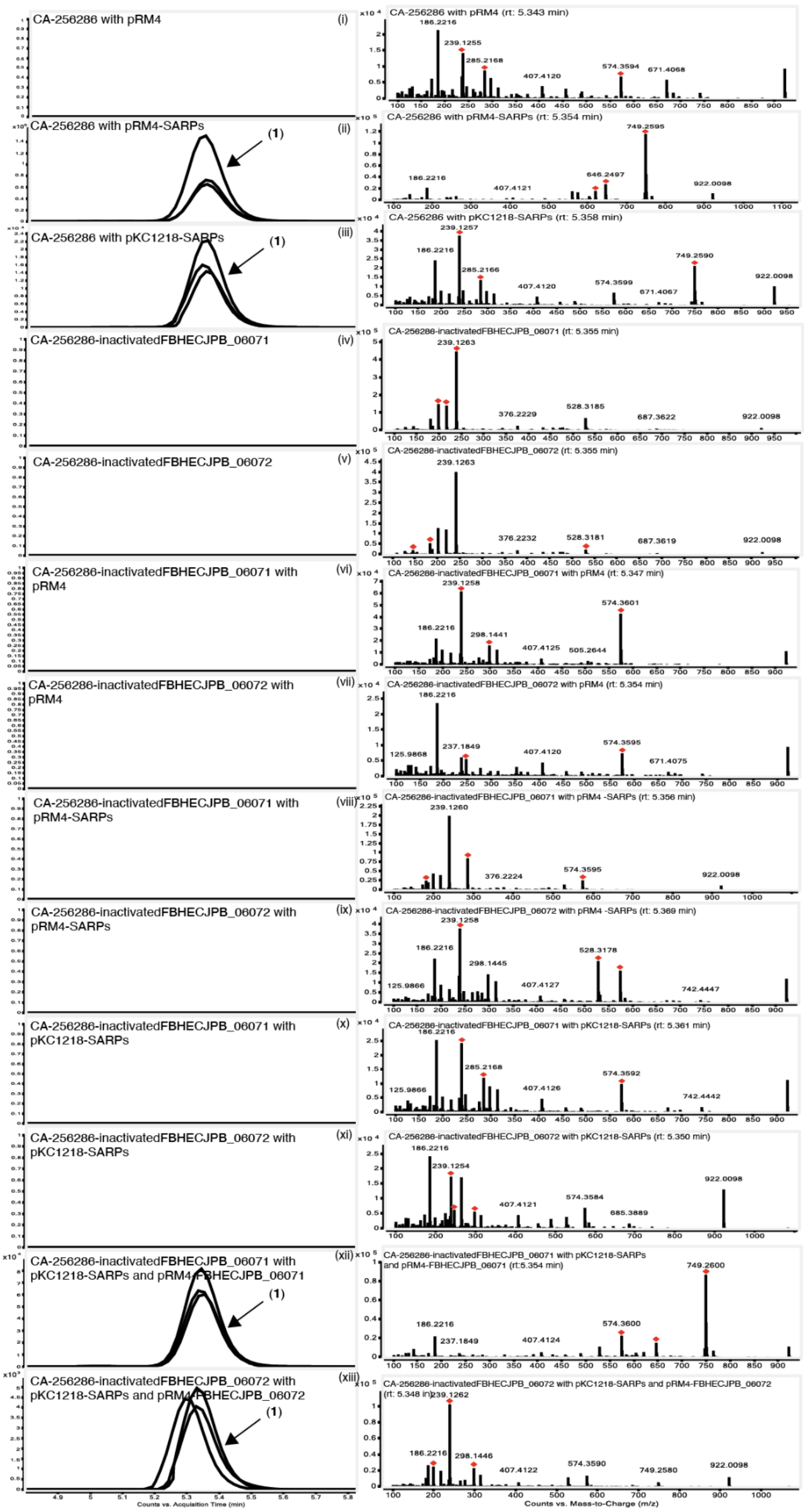

Figure S25: LC-MS analysis for the production of (**1**) in CA-256286 with pRM4 (i), CA-256286 with pRM4-SARPs (ii), CA-256286 with pKC1218-SARPs (iii), CA-256286-inactivatedFBHECJPB_06071 (iv), CA-256286-inactivatedFBHECJPB_06072 (v), CA-256286-inactivatedFBHECJPB_06071 with pRM4 (vi), CA-256286-inactivatedFBHECJPB_06072 with pRM4 (vii), CA-256286-inactivatedFBHECJPB_06071 with pRM4-SARPs (viii), CA-256286-inactivatedFBHECJPB_06072 with pRM4-SARPs (ix), CA-256286-inactivatedFBHECJPB_06071 with pKC1218-SARPs (x), CA-256286-inactivatedFBHECJPB_06072 with pKC1218-SARPs (xi), CA-256286-inactivatedFBHECJPB_06071 with pKC1218-SARPs and pRM4-FBHECJPB_06071 (xii) and CA-256286-

inactivatedFBHECJPB_06072 with pKC1218-SARPs and pRM4-FBHECJPB_06072(xiii). Replicates are displayed in overlaid mode. On the right from every EIC, a mass spectrum is extracted for approximate RT 5.35 min.

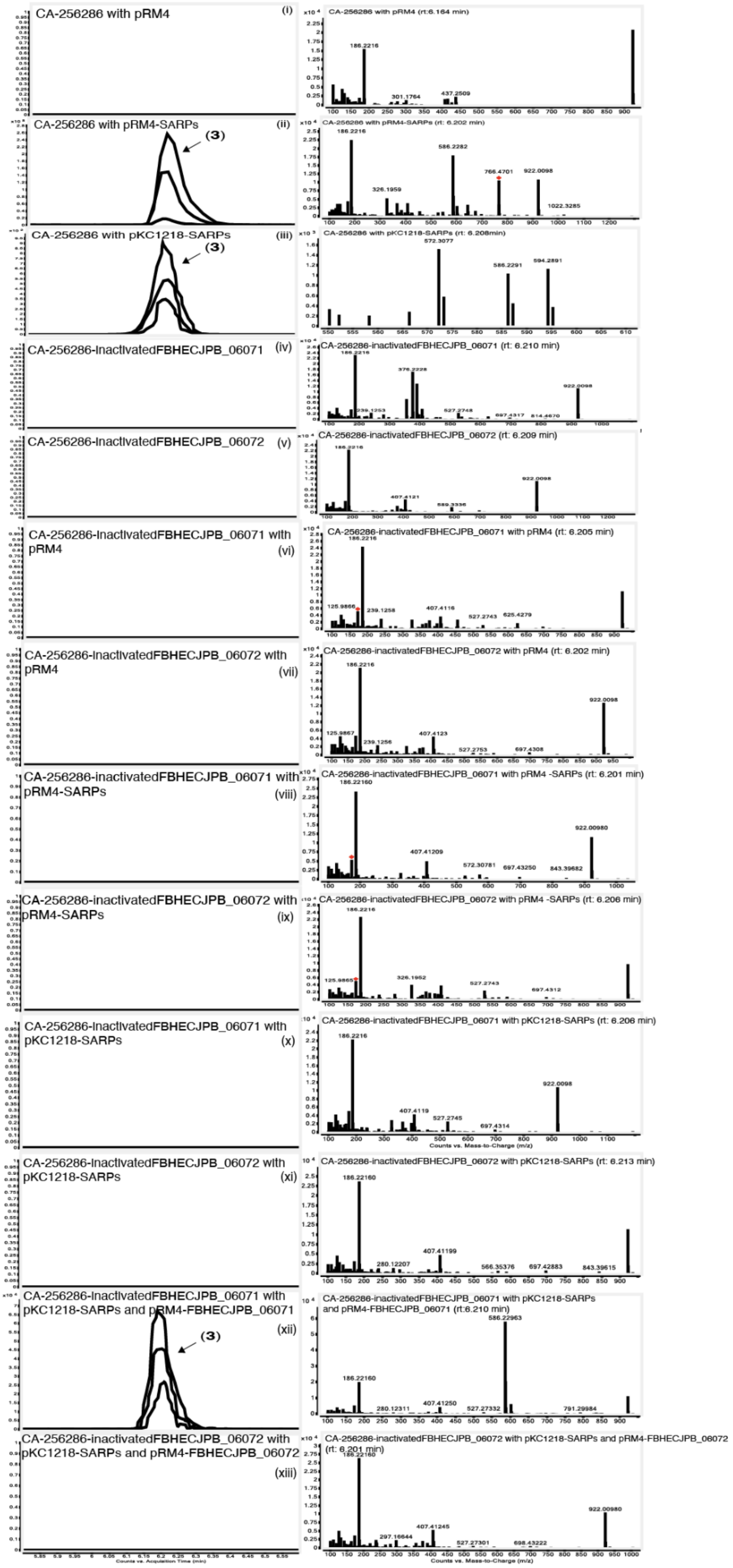

Figure S26: LC-MS analysis for the production of (**3**) in CA-256286 with pRM4 (i), CA-256286 with pRM4-SARPs

(ii), CA-256286 with pKC1218-SARPs (iii), CA-256286-inactivatedFBHECJPB_06071 (iv), CA-256286-inactivatedFBHECJPB_06072 (v), CA-256286-inactivatedFBHECJPB_06071 with pRM4 (vi), CA-256286-inactivatedFBHECJPB_06072 with pRM4 (vii), CA-256286-inactivatedFBHECJPB_06071 with pRM4-SARPs (viii), CA-256286-inactivatedFBHECJPB_06072 with pRM4-SARPs (ix), CA-256286-inactivatedFBHECJPB_06071 with pKC1218-SARPs (x), CA-256286-inactivatedFBHECJPB_06072 with pKC1218-SARPs (xi), CA-256286-inactivatedFBHECJPB_06071 with pKC1218-SARPs and pRM4-FBHECJPB_06071 (xii) and CA-256286-inactivatedFBHECJPB_06072 with pKC1218-SARPs and pRM4-FBHECJPB_06072(xiii). Replicates are displayed in overlaid mode. On the right from every EIC, a mass spectrum is extracted for approximate RT 6.20 min.

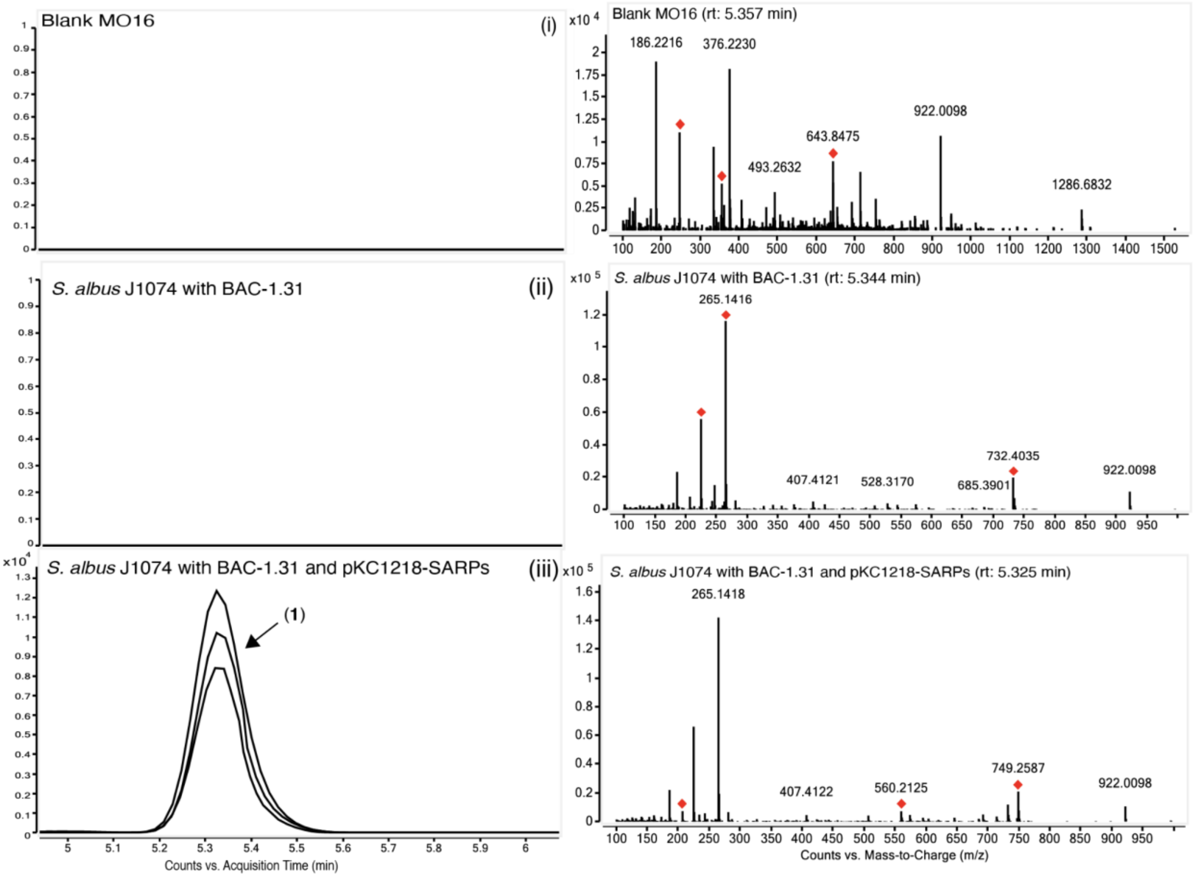

Figure S27: LC-MS analysis for the production of (**1**) in *S. albus* J1074 with BAC-1.31 (ii) and *S. albus* J1074 with BAC-1.31 and pKC1218-SARPs (iii). Chromatogram (i) represents blank samples from MO16 medium (negative control). Replicates are displayed in overlaid mode. On the right from every EIC, a mass spectrum is extracted for approximate RT 5.35 min for one of the replicates.

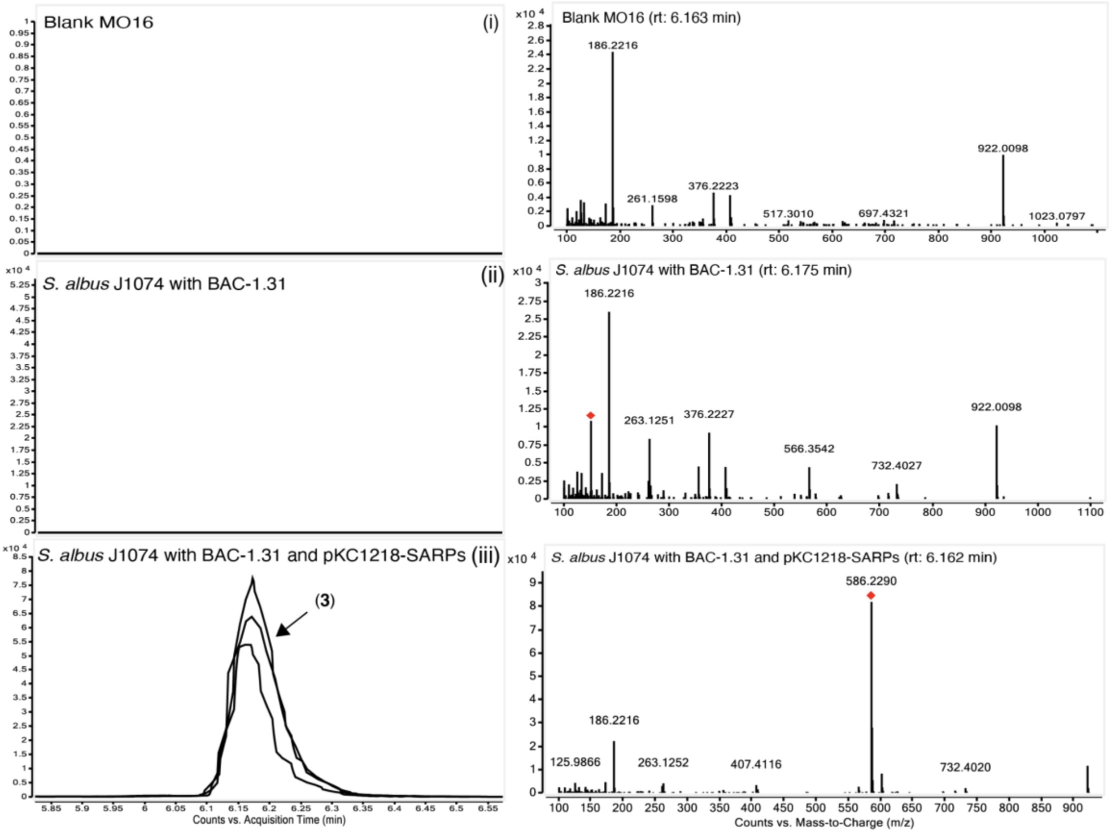

Figure S28: LC-MS analysis for the production of (**3**) in *S. albus* J1074 with BAC-1.31 (ii) and *S. albus* J1074 with BAC-1.31 and pKC1218-SARPs (iii). Chromatogram (i) represents blank samples from MO16 medium (negative control). Replicates are displayed in overlaid mode. On the right from every EIC, a mass spectrum is extracted for approximate RT 6.16 min for one of the replicates.

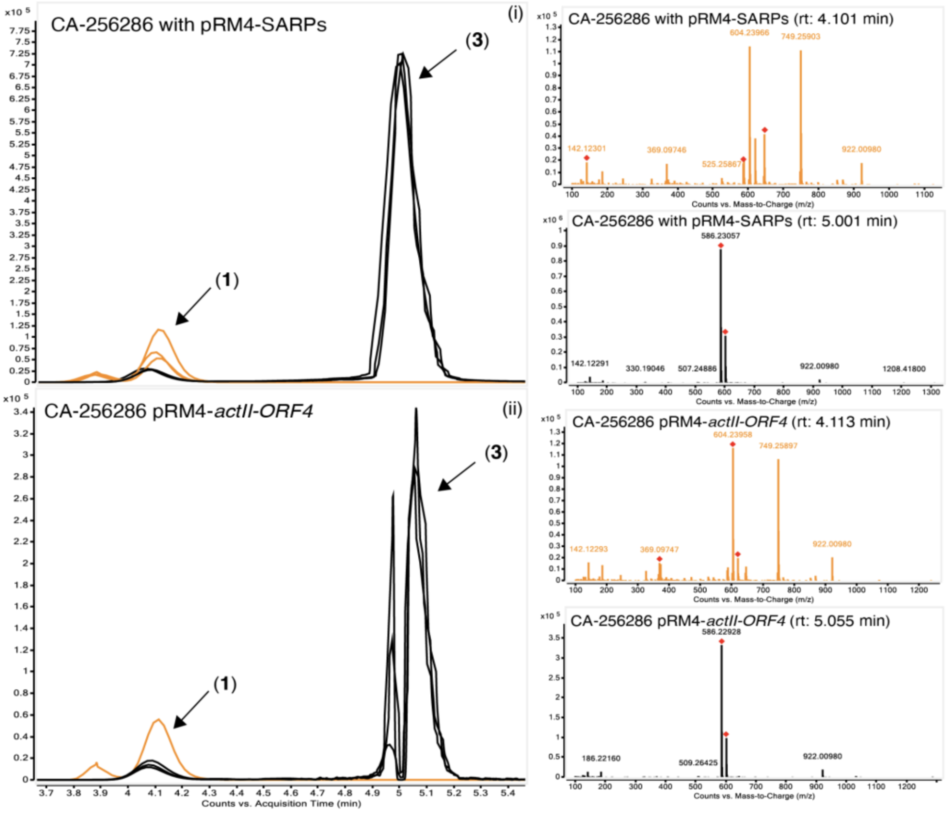

Figure S29: LC-MS analysis for the production of (**1**) and (**3**) in CA-256286 with pRM4-SARPs (i) and CA-256286 with pRM4-*actII-ORF4.* Replicates and the two different EICs are displayed in overlaid mode. On the right from every EIC, a mass spectrum is extracted for approximate RT 4.1 and 5.0 min for one of the replicates.

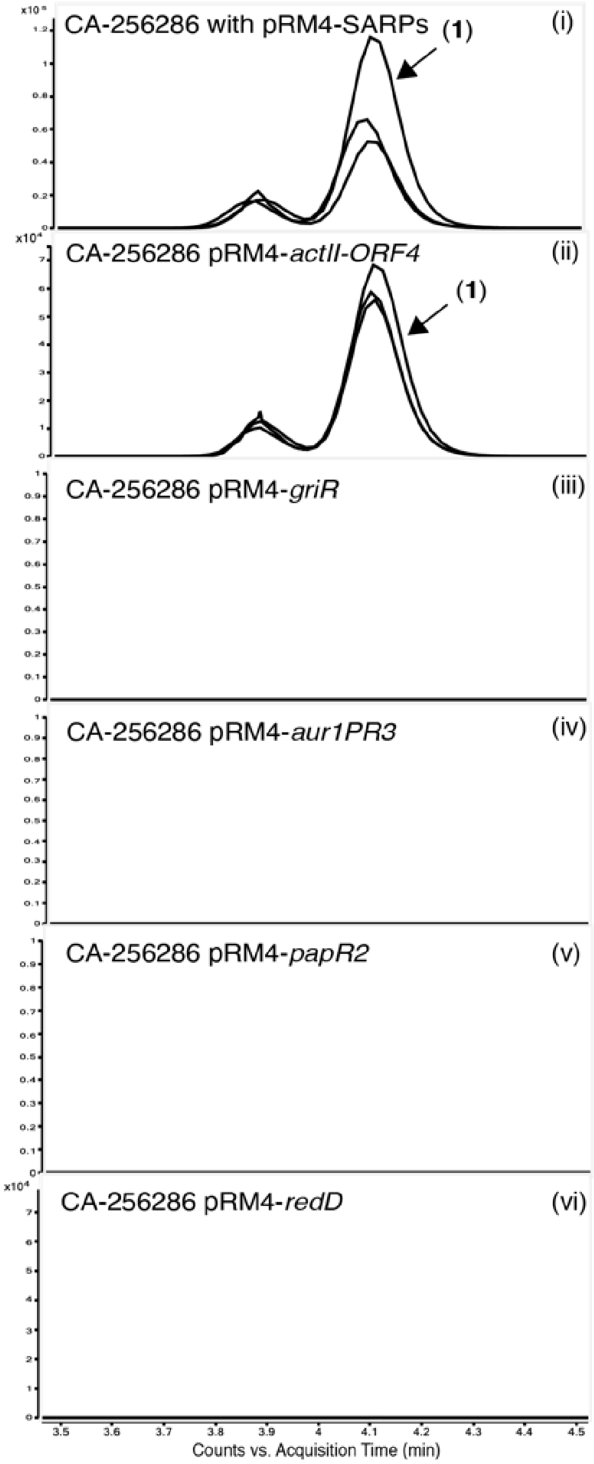

Figure S30: LC-MS analysis for the production of (**1**) in CA-256286 with pRM4-SARPs (i), CA-256286 with pRM4-*actII-ORF4* (ii), CA-256286 with pRM4-*griR* (iii), CA-256286 with pRM4-*aur1PR3* (iv), CA-256286 with pRM4-*papR2* (v) and CA-256286 with pRM4-*reD* (vi). Replicates are displayed in overlaid mode.

Supplementary information 7: BLAST analyses

Table S2: BLAST against the genome of *Streptomyces albidoflavus* J1074 (ASM35952v1) using detoxification genes from *Streptomyces coelicolor.*

**Gene name**	**Origin of the gene**	**Best BLAST hit score**	**Pairwise identity, %**	**E value**	**Position, nt**
mshA (SCO4204)	*S. coelicolor* A3(2)	1393.49<br><br>No other significant hits	82.2%	0	3,825,507 - 3,826,907
mshB (SCO5126)	*S. coelicolor* A3(2)	598.201<br><br>2 other hits, but of much lower quality	75.1%	4.95e-171	1,996,898 - 1,996,010
mshC (SCO1663)	*S. coelicolor* A3(2)	1512.51<br><br>143.752	87.4%<br><br>71.7%	0<br><br>4.39e-34	5,860,358 - 5,861,583<br><br>2,935,694 - 2,935,432
mshD (SCO4151)	*S. coelicolor* A3(2)	825.426<br><br>One another hit, but of low quality	79.9%	0	3,750,284 - 3,749,361
Mca (SCO4967)	*S. coelicolor* A3(2)	1081.5<br><br>64.404	87.2%<br><br>71.8%	0<br><br>2.40e-10	4,590,064 - 4,590,945<br><br>1996877 - 1996754

Supplementary information 8: Biosynthesis of griseusins

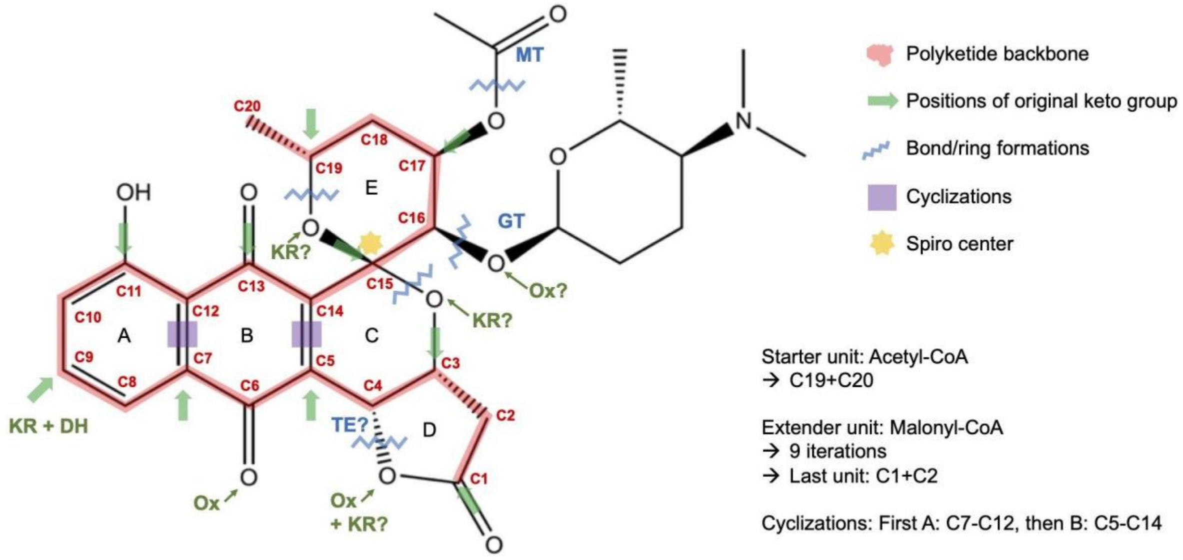

Figure S31: Schematic of proposed type II PKS features for biosynthesis of compound (**3**) 3'- O-α-D-forosaminyl-griseusin A. KR: Ketoreductase, DH: Dehydratase, Ox: Oxidation, MT: Methyltransferase, GT: Glycosyltransferase.

Supplementary information 9: BGC 1.31

Table S3: All genes from BGC 1.31 and their putative functions. The genes marked in green indicate genes we are convinced are involved, yellow indicates genes that might be involved and unmarked ones are unknown if they are involved. For each of the genes, it is registered whether the peptide was detected in proteomics analysis and whether the expression level was found to be significantly higher in strain *Streptomyces* sp. CA-256286 (pRM4-SARPs) than *Streptomyces* sp. CA-256286 (pRM4) in transcriptomics analysis.

**Locus tag Prokka**	**Locus tag NCBI**	**Nr.**	**Comment**	**antiSMASH predicted putative function**	**BLASTP top hit**	**Proteomics**	**Transcritptomics**
FBHECJPB_06042	JHY03_07500	1		SMC family ATPase	AAA family ATPase [Streptomyces sp. MBT58]		
FBHECJPB_06043	JHY03_07490	2		Lrp/AsnC family transcriptional regulator	MULTISPECIES: Lrp/AsnC family transcriptional regulator [Streptomyces]		X
FBHECJPB_06044	JHY03_07480	3	Sulfurtransferase	sulfurtransferase	MULTISPECIES: rhodanese-like domain-containing protein [Streptomyces]		X
FBHECJPB_06045	JHY03_07470	4		hypothetical protein	DUF885 domain-containing protein [Streptomyces sp. NRRL S-623]		X
FBHECJPB_06046	JHY03_07460	5		Lrp/AsnC family transcriptional regulator	MULTISPECIES: Lrp/AsnC family transcriptional regulator [Streptomyces]		X
FBHECJPB_06047	JHY03_07450	6		cystathionine gamma-synthase	MULTISPECIES: PLP-dependent transferase [Streptomyces]	X	X
FBHECJPB_06048	JHY03_07440	7		GNAT family N-acetyltransferase	MULTISPECIES: GNAT family N-acetyltransferase [Streptomyces]		X
FBHECJPB_06049	JHY03_07430	8		hypothetical protein	hypothetical protein [Streptomyces fulvissimus]		
FBHECJPB_06050	JHY03_07420	9		SWIM zinc finger family protein	MULTISPECIES: SWIM zinc finger family protein [Streptomyces]		
FBHECJPB_06051	JHY03_07410	10		alkaline phosphatase	MULTISPECIES: alkaline phosphatase D family protein [Streptomyces]		
FBHECJPB_06052	JHY03_07400	11		PLP-dependent aminotransferase family protein	aminotransferase class I/II-fold pyridoxal phosphate-dependent enzyme [Streptomyces fulvissimus]		X
FBHECJPB_06053	JHY03_07390	12		hypothetical protein	hypothetical protein YUYDRAFT_03072 [Streptomyces sp. ScaeMP-e48]		
FBHECJPB_06054	JHY03_07380	13		alpha/beta fold hydrolase	MULTISPECIES: alpha/beta fold hydrolase [Streptomyces]		
FBHECJPB_06055	JHY03_07370	14		sigma-70 family RNA polymerase sigma factor	RNA polymerase sigma factor SigJ [Streptomyces sp. MBT57]		X
FBHECJPB_06056	JHY03_07360	15		GntR family transcriptional regulator	MULTISPECIES: GntR family transcriptional regulator [Streptomyces]	X	
FBHECJPB_06057	JHY03_07350	16		ROK family transcriptional regulator	ROK family protein [Streptomyces luridiscabiei]		X

(Table continued)

FBHECJPB_06058	JHY03_07340	17		hypothetical protein	MULTISPECIES: hypothetical protein [Streptomyces]		
FBHECJPB_06059	JHY03_07330	18		G/U mismatch-specific DNA glycosylase	MULTISPECIES: G/U mismatch-specific DNA glycosylase [Streptomyces]		
FBHECJPB_06060	JHY03_07320	19		adenylosuccinate lyase	adenylosuccinate lyase [Streptomyces microflavus]	X	X
FBHECJPB_06061	JHY03_07310	20		TetR family transcriptional regulator	MULTISPECIES: TetR family transcriptional regulator [Streptomyces]		
FBHECJPB_06062 / qor	JHY03_07300 / qor	21	Oxidureductase	NADPH:quinone reductase-dependent oxidoreductase	zinc-binding dehydrogenase [Streptomyces sp. 2R]	X	X
FBHECJPB_06063	JHY03_07290	22	Exporter	export protein	MULTISPECIES: multidrug efflux MFS transporter [Streptomyces]		X
FBHECJPB_06064	JHY03_07280	23	KR	sorbitol utilization protein SOU2	MULTISPECIES: SDR family oxidoreductase [Streptomyces]	X	X
FBHECJPB_06065	JHY03_07270	24	Glycosyl transferase	glycosyl transferase family protein	MULTISPECIES: DUF1205 domain-containing protein [Streptomyces]	X	X
FBHECJPB_06066	JHY03_07260	25		hypothetical protein	MULTISPECIES: hypothetical protein [Streptomyces]		X
FBHECJPB_06067 / desVI	JHY03_07250 / desVI	26	Methyltransferase Similar to SpnS dimethyltransferase	dTDP-3-amino-3,4, 6-trideoxy-alpha-D-glucopyranose	MULTISPECIES: class I SAM-dependent methyltransferase [Streptomyces]	X	X
FBHECJPB_06068 / rfbA	JHY03_07240 / rfbA	27	Thymidylyltransferase	glucose-1-phosphate thymidylyltransferase	MULTISPECIES: glucose-1-phosphate thymidylyltransferase RfbA [Streptomyces]		X
FBHECJPB_06069	JHY03_07230	28	Clyclase CYC C7-C12	actinorhodin polyketide synthase bifunctional cyclase/dehydratase	hypothetical protein BEH93_30180 [Streptomyces sp. 2R]	X	X
FBHECJPB_06070	JHY03_07220	29	ACP	acyl carrier protein	MULTISPECIES: acyl carrier protein [Streptomyces]	X	X
FBHECJPB_06071	JHY03_07210	30	Core type II PKS	ketosynthase chain-length factor	MULTISPECIES: ketosynthase chain-length factor [unclassified Streptomyces]	X	X
FBHECJPB_06072	JHY03_07200	31	Core type II PKS	beta-ketoacyl-[acyl-carrier-protein] synthase family protein	MULTISPECIES: beta-ketoacyl-[acyl-carrier-protein] synthase family protein [Streptomyces]	X	X
FBHECJPB_06073	JHY03_07190	32	Thioesterase	thioesterase	MULTISPECIES: thioesterase [Streptomyces]	X	X
FBHECJPB_06074	JHY03_07180	33	Oxidureductase	NADPH-dependent oxidoreductase	MULTISPECIES: NAD(P)H-dependent oxidoreductase [Streptomyces]		X

(Table continued)

FBHECJPB_06075	JHY03_07170	34		hypothetical protein	MULTISPECIES: 4'-phosphopantetheinyl transferase superfamily protein [unclassified Streptomyces]		X
FBHECJPB_06076	JHY03_07160	35	Similar to SpnO dehydrase	NDP-hexose 2,3-dehydratase	MULTISPECIES: NDP-hexose 2,3-dehydratase family protein [unclassified Streptomyces]		X
FBHECJPB_06077	JHY03_07150	36	Similar to SpnN ketoreductase	Gfo/Idh/MocA family oxidoreductase	Gfo/Idh/MocA family oxidoreductase [Streptomyces sp. 2R]	X	X
FBHECJPB_06078 / rfbH	JHY03_07140 / rfbH	37	Similar to SpnQ dehydrase	lipopolysaccharide biosynthesis protein RfbH	MULTISPECIES: lipopolysaccharide biosynthesis protein RfbH [Streptomyces]	X	X
FBHECJPB_06079	JHY03_07130	38	Cyclase CYC C5-C14	cyclase	MULTISPECIES: MBL fold metallo-hydrolase [Streptomyces]		X
FBHECJPB_06080	JHY03_07120	39	SARP	regulatory protein	MULTISPECIES: AfsR/SARP family transcriptional regulator [Streptomyces]	X	X
FBHECJPB_06081	JHY03_07110	40		hydrolase	MULTISPECIES: acyl-CoA dehydrogenase family protein [Streptomyces]	X	X
FBHECJPB_06082	JHY03_07100	41	CYC C1-C18|C2-C19 (does not fit)	nuclear transport factor 2 family protein	MULTISPECIES: nuclear transport factor 2 family protein [Streptomyces]	X	X
FBHECJPB_06083	JHY03_07090	42	Oxidureductase	luciferase family protein	MULTISPECIES: LLM class flavin-dependent oxidoreductase [Streptomyces]		X
FBHECJPB_06084 / hcaB	JHY03_07080 / hcaB_	43	KR	3-phenylpropionate-dihydrodiol/cinnamic acid-dihydrodiol dehydrogenase	MULTISPECIES: SDR family oxidoreductase [Streptomyces]	X	X
FBHECJPB_06085	JHY03_07070	44	Acyltransferase	acyltransferase	MULTISPECIES: acyltransferase [unclassified Streptomyces]		X
FBHECJPB_06086	JHY03_07060	45	Oxidureductase / KR	short chain dehydrogenase	MULTISPECIES: SDR family NAD(P)-dependent oxidoreductase [Streptomyces]	X	X
FBHECJPB_06087	JHY03_07050	46	Cyclase CYC C7-C12 (similar to S. griseus cyclase)	actinorhodin polyketide synthase bifunctional cyclase/dehydratase	SRPBCC family protein [Streptomyces sp. 2R]	X	X
FBHECJPB_06088	JHY03_07040	47	KR (similar to S. griseus KR)	SDR family NAD(P)-dependent oxidoreductase	MULTISPECIES: 3-oxoacyl-ACP reductase FabG [Streptomyces]	X	X
FBHECJPB_06089	JHY03_07030	48	Aminotranferase, similar to SpnR transaminase	DegT/DnrJ/EryC1/StrS family aminotransferase	MULTISPECIES: DegT/DnrJ/EryC1/StrS family aminotransferase [Streptomyces]	X	X
FBHECJPB_06090	JHY03_07020	49	SARP	transcriptional regulator	AfsR/SARP family transcriptional regulator [Streptomyces sp. 2R]	X	X
FBHECJPB_06091 / rfbB	JHY03_07010 / rfbB	50	Dehydratase	dTDP-glucose 4,6-dehydratase	MULTISPECIES: dTDP-glucose 4,6-dehydratase [Streptomyces]	X	X

(Table continued)

FBHECJPB_06092	JHY03_07000	51		SGNH/GDSL hydrolase family protein	MULTISPECIES: SGNH/GDSL hydrolase family protein [unclassified Streptomyces]		X
FBHECJPB_06093	JHY03_06990	52		HlyC/CorC family transporter	MULTISPECIES: HlyC/CorC family transporter [Streptomyces]		
FBHECJPB_06094	JHY03_06980	53		HlyC/CorC family transporter	DUF21 domain-containing protein [Streptomyces sp. MBT58]		
FBHECJPB_06095	JHY03_06970	54		GNAT family N-acetyltransferase	MULTISPECIES: GNAT family N-acetyltransferase [Streptomyces]		
FBHECJPB_06096	JHY03_06960	55		toxin-antitoxin system HicB family antitoxin	MULTISPECIES: hypothetical protein [Streptomyces]		
FBHECJPB_06097	JHY03_06950	56		hypothetical protein	MULTISPECIES: hypothetical protein [Streptomyces]		
FBHECJPB_06098	JHY03_06940	57	Methyltransferase	class I SAM-dependent methyltransferase	methyltransferase domain-containing protein [Streptomyces sp. MBT58]		
FBHECJPB_06099	JHY03_06930	58		ATP-dependent dethiobiotin synthetase BioD	ATP-dependent dethiobiotin synthetase BioD [Streptomyces sp. MBT58]		
FBHECJPB_06100	JHY03_06920	59	SAM transaminase	adenosylmethionine--8-amino-7-oxononanoate transaminase	adenosylmethionine--8-amino-7-oxononanoate transaminase [Streptomyces sp. MBT58]	X	
FBHECJPB_06101	JHY03_06910	60		biotin synthase	biotin synthase BioB [Streptomyces sp. NRRL S-623]	X	
FBHECJPB_06102	JHY03_06900	61		8-amino-7-oxononanoate synthase	MULTISPECIES: 8-amino-7-oxononanoate synthase [Streptomyces]		X
FBHECJPB_06103	JHY03_06890	62		hypothetical protein	MULTISPECIES: DUF397 domain-containing protein [Streptomyces]		X
FBHECJPB_06104	JHY03_06880	63		XRE family transcriptional regulator	DNA-binding protein [Streptomyces fulvissimus DSM 40593]		X
FBHECJPB_06105	JHY03_06870	64		ATP-binding protein	MULTISPECIES: ATP-binding protein [Streptomyces]		
FBHECJPB_06106	JHY03_06860	65		LysR family transcriptional regulator	MULTISPECIES: LysR family transcriptional regulator [Streptomyces]		
FBHECJPB_06107	JHY03_06850	66		LysE family translocator	MULTISPECIES: LysE family translocator [Streptomyces]		X
FBHECJPB_06108	JHY03_06840	67		NlpC/P60 family protein	MULTISPECIES: C40 family peptidase [Streptomyces]		X
